# PlatROB: An open-source, modular, and low-cost hardware platform for mobile robotics and AI education

**DOI:** 10.1016/j.ohx.2026.e00747

**Published:** 2026-02-07

**Authors:** Jose Balbuena, Julio Sinche, Diego Quiroz, Diego Arce, Elizabeth Villota

**Affiliations:** Department of Engineering, Pontificia Universidad Católica del Perú, Av. Universitaria 1801, San Miguel, 15088 Lima, Peru

**Keywords:** Open-source robotics platform, Mobile robot, Modular platform, Low-cost design, Locomotion Configuration, Educational evaluation

## Abstract

This paper introduces PlatROB, an open-source, modular, and low-cost educational robotics platform designed to facilitate hands-on learning in robotics and AI through system integration. PlatROB comprises four 3D-printable, classroom-ready modules: an Ackermann Drive Module (ADM), an Omnidirectional/Differential Drive Module (ODM/DDM), a Control and Processing Module (CPM) with NVIDIA Jetson Nano, and a 4-DoF Articulated Manipulation Module (AMM). Inter-module communication uses standardized I2C over DB9 connectors, integrating Arduino microcontrollers, motor drivers, encoders, IMUs, and ultrasonic sensors. Module costs range from $129 to $341. Performance validation shows the ADM supports 10 kg payload, achieves 25 cm turning radius and has 120 min autonomy (3 kg load), while the CPM sustains 40–100 min operation depending on neural-network workloads. The AMM provides 450 g payload capacity for introductory manipulation tasks. Successfully deployed in workshops and university courses with over 160 learners, demonstrating significant learning gains (p < 0.05) across academic levels and enabling teleoperation and autonomous navigation projects. All mechanical and electronic design files, build manuals, and validation code are open-source to support replication in resource-constrained settings. By providing a scalable, documented hardware-software stack compatible with ROS/ROS2, PlatROB lowers barriers to experiential learning in SLAM, perception, and advanced autonomy.

## Graphical abstract

**Figure:**
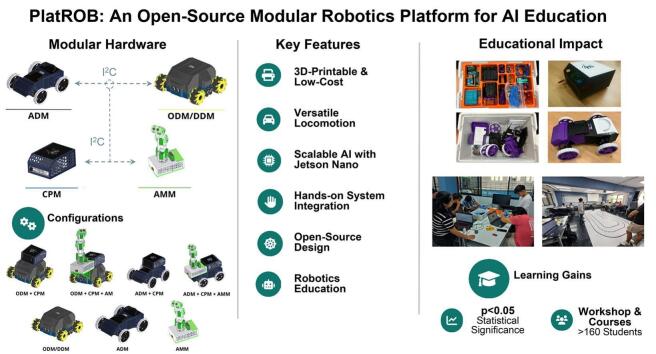

Specifications table

**Table:** 

**Hardware name**	PlatROB
**Subject area**	Educational tools and open source alternatives to existing infrastructure
**Hardware type**	Robotics
**Closest commercial ana- log**	Limo, developed by AgileX Robotics, is a commercial alternative due to its ability to switch locomotion systems. However, its high cost and the option to purchase only specific modules can limit its acquisition and widespread use.
**Open source license**	Non-commercial license: CC-BY 4.0
**Cost of hardware**	• Ackermann Drive Module (ADM): 568 PEN (150 USD) • Omnidirectional/Differential Drive Module (ODM/DDM): 735 PEN (193 USD) • Control and Processing Module (CPM): 1297 PEN (341 USD) • Articulated Manipulation Module (AM): 490 PEN (129 USD)
**Source file repository**	https://doi.org/10.17605/OSF.IO/KU92V

## 1 Hardware in context

The growing demand for robotics and artificial intelligence (AI) education necessitates accessible, modular, and affordable hardware platforms that enable hands-on learning of system integration, autonomous navigation, and real-world problem-solving [1], [2]. While numerous educational robots exist, most exhibit limitations in configurability, cost, or scalability, restricting their adaptability across diverse curricula. Low-cost platforms like Mona (£100, 45 g) [3] and HeRo ($18, 25 cm/s max speed) [4] offer entry-level accessibility but lack advanced perception or processing capabilities. Conversely, feature-rich platforms such as ROMR ($1500, 90 kg payload) [5], Robotont 3 (€1500, 3.3 kg) [6] support ROS-based autonomy but incur higher costs, limiting institutional deployment. Modular designs like Flower∞Bots (<$50) [7] and Miniskybot (€57, 3-hour print time) [8] enhance customization but often sacrifice robustness or sensor integration.

Cost-effectiveness remains critical to make robotics education more accessible, with open-source hardware solutions leveraging 3D printing and off-the-shelf electronics to reduce expenses. Platforms such as SMARTmBOT ($210, 8 × ToF sensors) [9] and OpenScout (<$350, 15 kg payload) [10], and ESP32-based robots (<€50, AR-designed) [11] demonstrate that Arduino/Raspberry Pi ecosystems can maintain functionality while achieving sub-$350 budgets [2], [12].

Modularity is the key in modern educational robots, enabling scalable learning experiences to suit diverse student needs and curricula. Platforms such as MBot supports configurations from basic models (<$100) to advanced versions with Raspberry Pi/Jetson Nano ($350) [13], while Robotont 3′s consolidated PCB simplifies assembly [6]. Systems supporting ≥12 expansion modules [14] or magnetically interlocking components (e.g., eSMAC kit [15]) allow progressive complexity without hardware redesign. This adaptability allows students to start with fundamental concepts and progressively tackle more complex projects.

Locomotion versatility is also essential for comprehensive mobile robotics kinematics education. Configurable drive systems, including differential/tracked/omnidirectional wheels [16], ExoMy’s triple-bogie suspension and Ackerman/crab steering ($400) [17], and Mecanum-based platforms (±6.02 cm error) [18] enable comparative mobility studies. ReMoRo’s three-wheel omnidirectional system (linear speed <32 cm/s) [19] further exemplifies this capability.

Developing system integration skills, understanding the interplay of hardware and software becomes a core objective in robotics education. Open-source platforms are instrumental in this, often incorporating widely used processors, sensors and actuators, and software frameworks such as ROS. For example, ROMR is a ROS-based platform that integrates a Nvidia Jetson Nano and an Arduino Mega, equipped with sensors like RPlidar A2 (16 m range, 10 Hz) and Intel Realsense cameras [5]. SMARTmBOT also utilizes ROS2 with a Raspberry Pi 4 and 8 ToF sensors [9]. The R2P framework even employs a CAN-Bus for real-time communication between STM32 Cortex-M3 based modules [20].

Most existing platforms focus on carrying lightweight, modular payloads such as sensors (e.g., ultrasonic, infrared, LiDAR, cameras, IMUs), microcontrollers, and communication modules for educational and research tasks like navigation, mapping, and swarm robotics. Notable exceptions include OpenScout (15 kg), ROMR (90 kg), and OPIL (Anda AGV, 400 kg), which are designed for heavier payloads, including packages for delivery or industrial logistics. Payloads are typically tailored to support hands-on learning in robotics and AI, emphasizing modularity and sensor integration. However, the ReMoRo platform is the only one identified that explicitly describes a robotic platform with an articulated arm, specifically a 3-DOF (degrees of freedom) cylindrical manipulator with a 1-DOF gripper.

Several robotics platforms have demonstrated their educational value on student learning and engagement through deployment in academic settings. The MBot platform, for example, has been used to train over 1,400 students across multiple courses since 2014 [13]. The DIY construction system by [21] was evaluated with 86 students and 35 teachers, showing its utility in teaching engineering and programming. Similarly, the Thymio robot, with a production cost under $39, has been adopted from kindergarten to university level [22], and workshops with the Cozmo robot framework have shown its effectiveness in fostering engagement [23]. The systematic review by [1] further confirms that robotics-based learning enhances student motivation and practical skills.

This paper presents PlatROB, an open-source, 3D-printable educational platform developed at PUCP designed for hands-on learning at diverse academic levels, with estimated costs of <$200 for the mobile base, <$130 for the robotic-arm and <$350 for the processing unit. PlatROB’s modular architecture enables: i) interchangeable locomotion (Ackermann steering, differential drive, and omnidirectional Mecanum wheels); ii) scalable computation using Arduino-based microcon- trollers and an NVIDIA Jetson Nano; iii) teleoperation for immediate classroom use, iv) extensible sensing via mounts for LiDAR, cameras and ultrasonic arrays; v) accessibility through predominantly 3D-printed components and cost-effective electronics; vi) flexibility afforded by a modular design that accommodates varied control modules, sensors, and actuators to tailor learning pathways; and vii) development of system-level understanding by allowing students to integrate hardware and software for activities ranging from teleoperation to autonomous behaviors using computer vision on the NVIDIA Jetson. Validated in workshops with mechatronics students, PlatROB aligns with constructivist learning principles through customizable hardware [11], enabling beginners to assemble 3D-printed vehicle mechanics and pre-soldered electronics in < 3 h while advanced users implement AI algorithms [24]. Its design philosophy draws from successes like MBot (1,400 + students trained) [13]. The paper covers hardware and software architecture, assembly and operation guidelines, validation and performance characterization, and educational evaluation, establishing PlatROB as a flexible, cost-effective solution for broadening access to robotics and AI education.

## 2 Hardware description

### 2.1 System overview

**Fig. 1:**
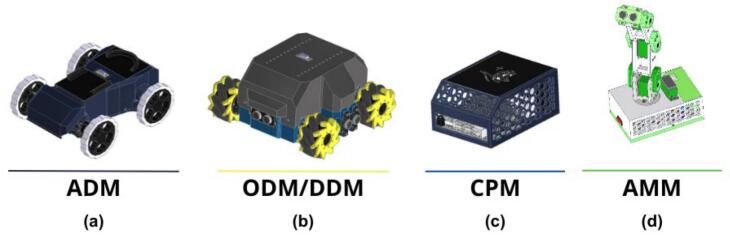
PlatROB modules. a) Ackermann Drive Module (ADM). b) Omnidirectional/Differential Drive Module (ODM/DDM). c) Control and Processing Module (CPM). d) Articulated Manipulation Module (AMM).

PlatROB comprises multiple interconnected modules crafted for educational robotic systems, each offering unique functionalities. These modules include the the Ackermann Drive Module (ADM), Omnidirectional/Differential Drive Module (ODM/DDM), the Control and Processing Module (CPM), and the Articulated Manipulation Module (AMM), as shown in Fig. 1. Each module has distinct characteristics that enhance the others’ capabilities. The subsequent sections explore their collaborative functionality, potential configurations, and key features that set this system apart from commercial competitors.

### 2.2 Design requirements

PlatROB was developed as a durable and versatile educational platform tailored to mobile robotics and AI instruction. Its design adhered to the requirements outlined in Table 1. By adopting a modular and 3D-printable architecture, PlatROB achieves an optimal balance of accessibility, adaptability, and learning depth, making it ideally suited for educational applications in mobile robotics and AI.

**Table 1:** PlatROB design requirements and descriptions.

**Accessibility and affordability**	Predominantly 3D-printable architecture and off-the-shelf components to enable cost-effective replication and broad adoption in teaching laboratories.
**Locomotion versatility**	Support for multiple ground-vehicle configurations (Ackermann steering, differential drive, and omnidirectional Mecanum) using common chassis elements and interchangeable wheels/hubs, enabling comparative kinematics activities.
**Compact form factor and portability**	All modules fit within a 30 × 30 × 15 cm envelope to simplify storage, transport, and classroom deployment.
**Cost-effectiveness**	Component selection and design-for-manufacture (DFM) reduce material usage, print time, and assembly time without compromising educational functionality.
**Standardized interconnection**	A common electrical and physical interface across modules (I2C bus over DB9 connectors) to ensure plug-and-play integration and simplify debugging.
**Robustness and maintainability**	Use of readily replaceable printed parts and standard fasteners; tolerance-aware printed fea- tures; modular subassemblies for rapid service and iteration in a classroom setting.
**Safety for classroom use**	Clear wiring and power guidelines, enclosure of moving parts where feasible, and operating procedures that minimize hazards.
**Extensible sensing and actuation**	Mechanical mounts and electrical headers for common sensors (e.g., ultrasonic, IMU; optional camera/LiDAR) and straightforward addition of actuators (e.g., servos).
**Scalable computation**	Compatibility with Arduino-based microcontrollers for low-level control and an NVIDIA Jetson Nano for on-board computer vision and higher-level behaviors.
**Educational alignment**	Learning activities that progress from assembly and teleoperation to basic autonomy and system integration, supporting varied prior experience levels and course formats.
**Performance targets (module-specific)**	ADM: small turning radius and classroom-appropriate speed; CPM: autonomy sufficient for lab sessions under typical workloads; AMM: payload and angular position adequate for sensor handling and introductory manipulation.

### 2.3 Modular architecture overview

PlatROB employs a modular architecture designed to encompass a diverse range of mobile robot configurations. Each module incorporates specific features designed to improve student learning in these domains. A detailed summary of the features of each module, estimated cost and educational purpose is detailed in Table 2.

**Table 2:** Module description and characteristics.

Module	**Description**	**Features**	**Educational purpose**	**Cost (PEN)**	**Cost (USD)**
ADM	Ackermann Drive Module	Mobile robot with Ackermann configuration driven by microcontroller	Operation of mobile robots with Ackermann configuration − Inverse and direct mobile kinematic models − Wheel speed control	568	150
ODM/DDM	Omnidirectional/Differential Drive Module	Reconfigurable mobile robot microcontroller-driven with omnidirectional and differential configuration	Operation of mobile robots with omnidirectional and differential drive configurations − Implementation of position estimation algorithms − Sensor fusion (Encoder − IMU − distance sensor)	735	193
CPM	Control and Processing Module	Control module with SBC features	Wireless Robot Operation Types − Computer Vision − Path Planning Algorithms	1297	341
AMM	Articulated Manipulation Module	Robotic arm with 4 degrees of freedom with distance sensor and its own microcontroller	Inverse and direct kinematics models of arm − Implementation of Kalman filter in distance sensor − Control of robotic manipulators	490	129

**Fig. 2:**
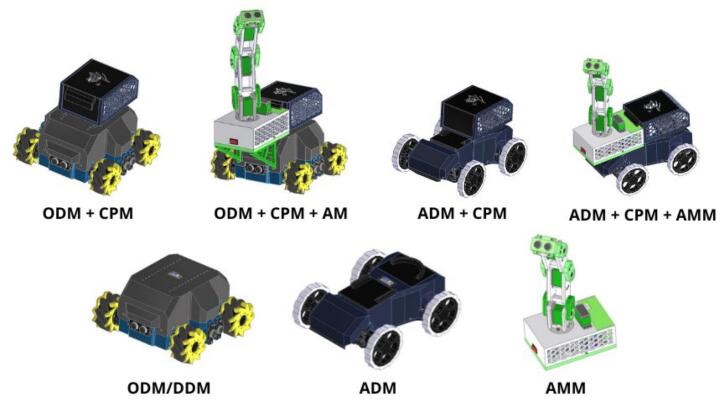
Various interconnections between PlatROB modules.

The modular design facilitates multiple kinematic configurations, enabling simulation of various real-world robotic applications. Fig. 2 illustrates the possible combinations that can be achieved with the four primary modules. For example, the Ackermann Drive Module (ADM) is well-suited for applications mirroring autonomous vehicles, such as delivery or transport robots. The Differential Drive Module (DDM) is typically employed in industrial or exploration robots that require precise maneuverability in constrained environments. The Omnidirectional Drive module (ODM) is appropriate for dynamic settings, such as automated warehouses or robots requiring multi-directional interaction. An Articulated Manipulation Module (AMM) is included to address the increasing relevance of manipulators in automated processes. Furthermore, the Central Processing Module (CPM), equipped with a Single Board Computer (SBC), supports advanced applications involving computer vision and machine learning. These modules can be interconnected in various configurations, including standalone ADM or ODM/DDM setups, or more complex assemblies incorporating the CPM and AMM, thus providing a highly adaptable and comprehensive educational platform.

**Fig. 3:**
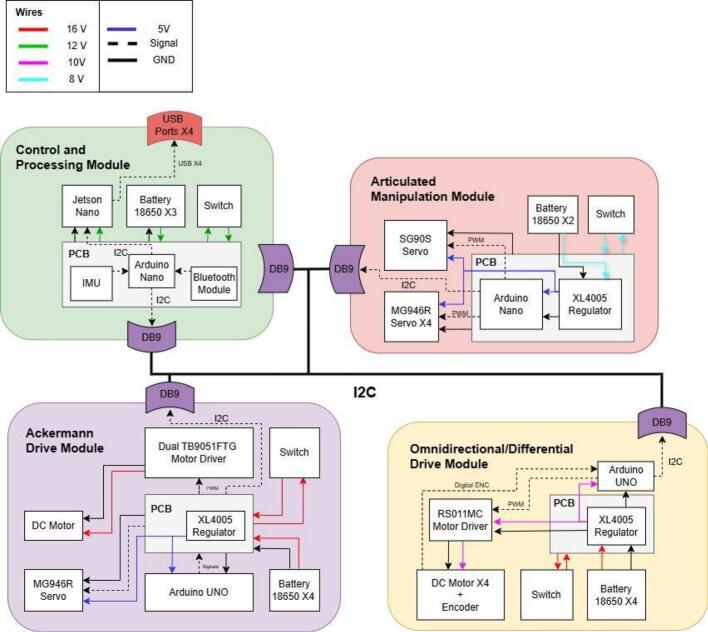
PlatROB’s hardware architecture for each module and I2C connection using DB9 connectors.

To streamline component procurement and assembly, the electronic hardware design of the PlatROB modules uses similar components, for example, DB9 connector, power-on relays, lithium batteries, and others. Consequently, the modules share a similar electronic schematic, as depicted in Fig. 3. Inter-module communication is facilitated by the I2C protocol, utilizing DB9 connectors for data transmission. Each module is equipped with at least one such connector to ensure interconnectivity across the platform.

**Fig. 4:**
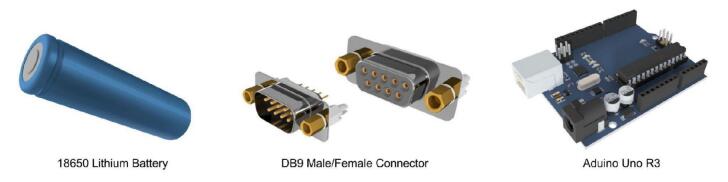
Shared electronic components across modules.

Among the standardized components, the Arduino Uno and Nano development boards served as the primary microcontroller in most modules. Its widespread adoption and ease of use allow students to rapidly familiarize themselves with the platform’s operation. Power is supplied to the modules via an arrangement of 18,650 lithium-ion batteries. This battery type was selected due to its cost-effectiveness and the ease with which individual cells can be replaced in the event of failure. Fig. 4 illustrates the shared electronic components across modules.

**Fig. 5:**
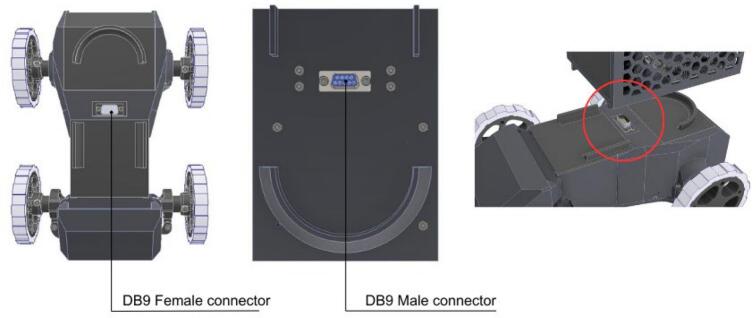
Mechanical coupling and assembly interface between the ADM and CPM.

Regarding mechanical assembly, the design prioritizes ease of reconfiguration by utilizing the mating force of the male and female DB9 connectors as the primary coupling mechanism. However, to ensure structural rigidity in demanding scenarios, the platform includes provisions for auxiliary screw fastening. Fig. 5 depicts a representative connection between the ADM and CPM, illustrating this physical interface.

### 2.4 Module descriptions

The subsequent sections will provide a detailed description of the components, costs, parts, and assemblies for each module comprising the PlatROB educational kit.

#### 2.4.1 Ackermann drive module (ADM)

**Fig. 6:**
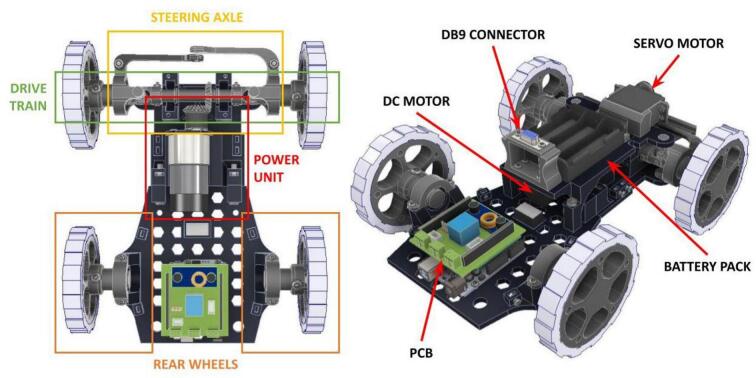
Ackermann Drive Module (ADM) main components. The left image defines the sub assemblies of the ADM and the right image details the main electronic components used in the ADM.

The ADM is a principal locomotion module for the PlatROB platform, designed to emulate the steering and movement dynamics characteristic of automotive vehicles. As illustrated in Fig. 6, the ADM comprises four wheels, a drivetrain, a power unit, and a steering axle. To simplify the mechanical implementation and reduce complexity, a front-wheel-drive configuration was adopted.

**Fig. 7:**
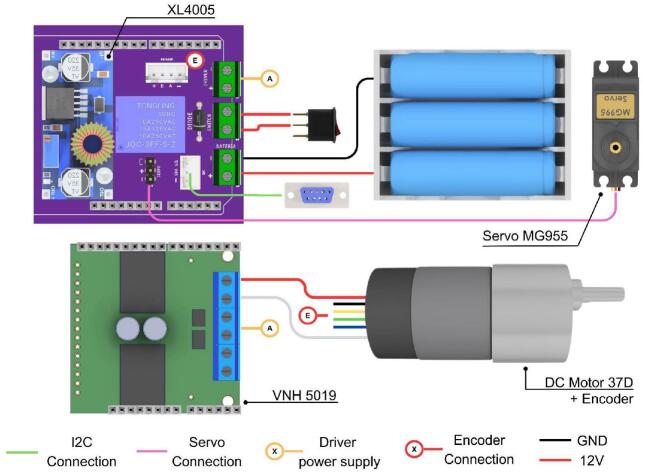
Custom designed PCB for the ADM module.

The electronic architecture of the ADM is depicted in Fig. 7, which shows the PCB and its interconnections with the various electronic components. The module incorporates a DC motor equipped with an integrated encoder, which, in conjunction with a motor driver and a conical gear arrangement, provides traction to the front wheels. Steering is achieved via a DC servomotor that actuates the steering axle, enabling directional control of the robot. Power for all actuators is supplied by a lithium-ion battery pack, regulated by a step-down voltage converter. A custom-designed PCB facilitates the integration and interconnection of all electronic components within the ADM.

**Fig. 8:**
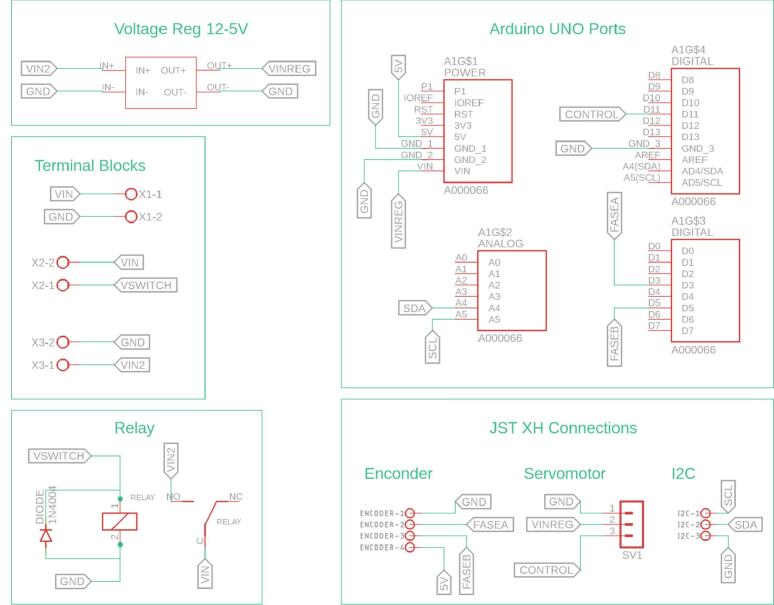
Detailed electronic schematic of the ADM module.

Fig. 8 details the internal connections of the ADM module’s PCB. To enhance legibility, components have been organized into specific functional groups. Notably, the Arduino Uno interface is segmented into distinct blocks corresponding to the physical location and type of its ports. Furthermore, the PCB incorporates a JST connector specifically designated to facilitate I2C communication between modules.

#### 2.4.2 Omnidirectional/differential drive module (ODM/DDM)

**Fig. 9:**
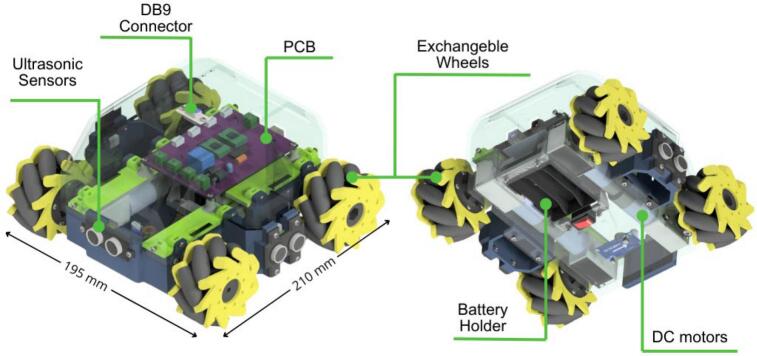
ODM/DDM main characteristics and dimensions.

This drive module is engineered around a compact, rectangular 3D-printed chassis with dimensions of 19.5 × 21 cm as shown in Fig. 9. It houses four 12 V DC brushed motors, each with an integrated right-angle gearbox. This specific motor and gearbox arrangement allows the motors to be aligned longitudinally within the module, a design choice that significantly reduces the overall footprint and creates internal space for mounting electronic components between the motors. Each motor is equipped with a quadrature encoder, enabling the implementation of closed-loop speed control and odometry algorithms crucial for autonomous navigation and precise positioning. The motor axles protrude from the chassis, facilitating the attachment of either standard wheels (for a differential drive configuration) or omnidirectional wheels. Connection is achieved using custom-designed, 3D-printed hubs secured with set screws. This design ensures straightforward wheel replacement and allows for a simple transition between omnidirectional and differential drive kinematics, enhancing the module’s versatility.

**Fig. 10:**
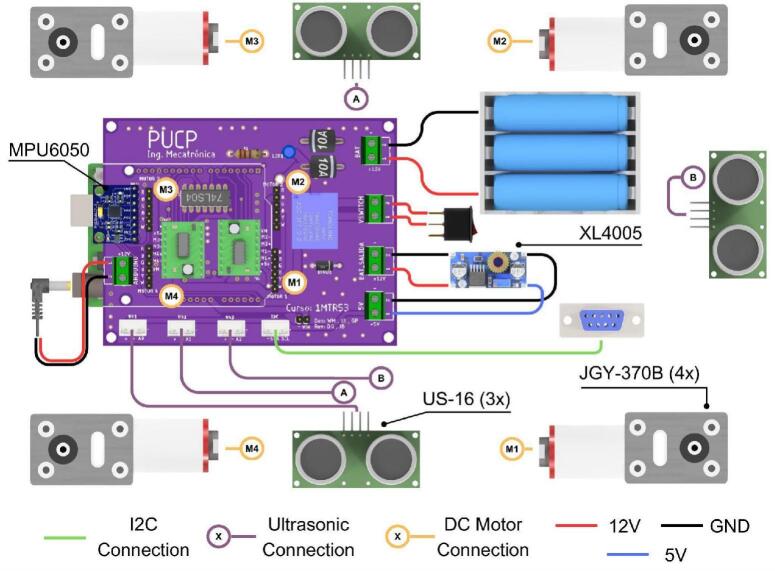
Custom designed PCB for the ODM/DDM module.

Power for the module is supplied by three 18,650 lithium-ion batteries, which are housed in a battery holder located on the underside of the chassis for convenient access and replacement. A rocker switch, in conjunction with a relay, controls the module’s power state. This configuration provides an opportunity for students to understand basic power circuit design and its application in robotics. Finally, a custom-designed PCB integrates an Arduino R3 microcontroller, three ultrasonic sensors for obstacle detection, an Inertial Measurement Unit (IMU) for orientation sensing, motor drivers, and interfaces for reading the motor encoders, as illustrated in Fig. 10.

**Fig. 11:**
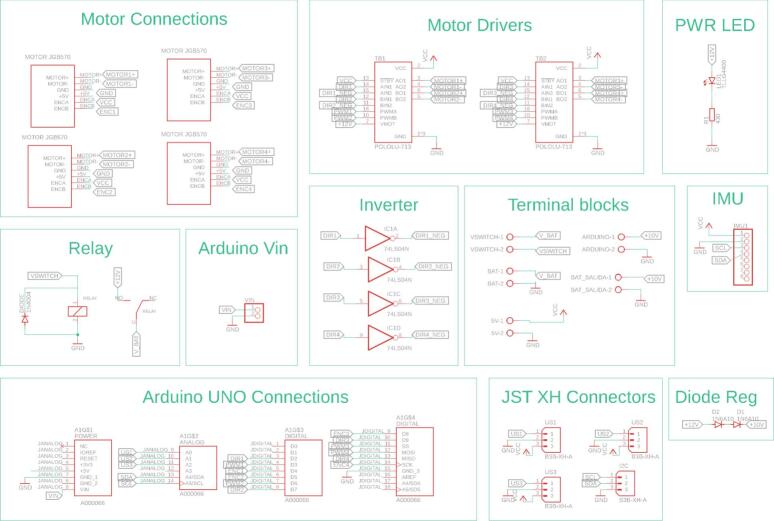
Detailed electronic schematic of the ODM/DDM module.

Fig. 11 presents the detailed internal schematic of the PCB, with components organized by functional group. The design incorporates several features specifically to streamline assembly, most notably the use of male and female pin headers for interfacing with the motors, IMU, and motor drivers. Furthermore, a logic inverter is integrated into the control circuitry. This optimizes pin usage by enabling motor direction control via two lines rather than the standard three, thereby conserving microcontroller resources.

#### 2.4.3 Control and Processing Module (CPM)

The Control and Processing Module (CPM) is engineered to provide students with practical experience utilizing SBCs, which are integral to advanced robotics. This module specifically incorporates an NVIDIA Jetson Nano, chosen for its robust processing capabilities, suitability for AI applications, and cost-effectiveness within an educational framework. The inclusion of the NVIDIA Jetson facilitates the integration of AI concepts, such as neural networks and computer vision, directly onto the hardware platform. Consequently, the CPM enables hands-on learning experiences in areas including wireless robot operation, implementation of computer vision algorithms, and development of path planning strategies.

**Fig. 12:**
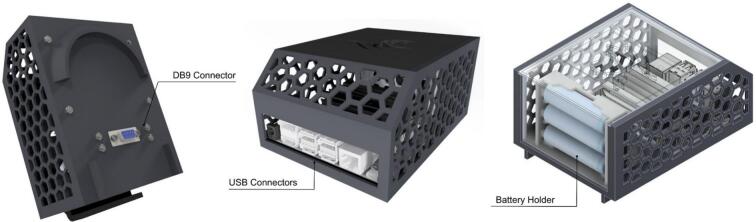
Illustrative views of the CPM module.

The NVIDIA Jetson supports the connection of diverse peripherals, such as 2D LiDAR, RGB cameras, depth sensors, and microphones. The CPM design, as shown in Fig. 12, ensures that the USB ports of the NVIDIA Jetson remain accessible, allowing for straightforward customization and peripheral integration. Furthermore, the module is equipped with a DB9 connector to facilitate standardized I2C communication with other modules within the PlatROB ecosystem.

In the mechanical design, a chassis was developed to support key components such as the NVIDIA Jetson and the batteries. The structure was conceived to prioritize easy access to connection ports and the electronic system as a whole, facilitating maintenance and integration. Additionally, the module is equipped with a removable cover, which provides adaptability to future requirements, such as the incorporation of a camera, LiDAR sensor, or other peripherals.

**Fig. 13:**
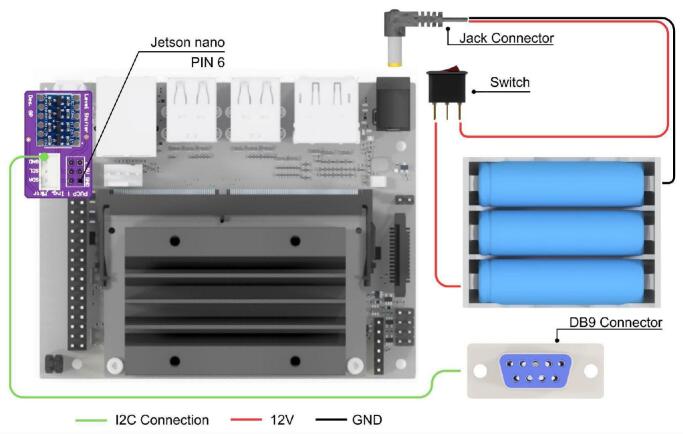
CPM hardware interfaces.

Regarding its electronic design, the CPM is powered by three 18,650 lithium-ion batteries, selected to provide adequate power for the NVIDIA Jetson. To enable communication with microcontroller-based modules (e.g., those using Arduino), a bidirectional logic level shifter is employed. This component translates the 3.3 V logic levels of the Jetson Nano to the 5 V logic levels utilized by the Arduino microcontrollers, ensuring reliable I2C data exchange. A detailed schematic illustrating the system connections within the CPM is presented in Fig. 13.

**Fig. 14:**
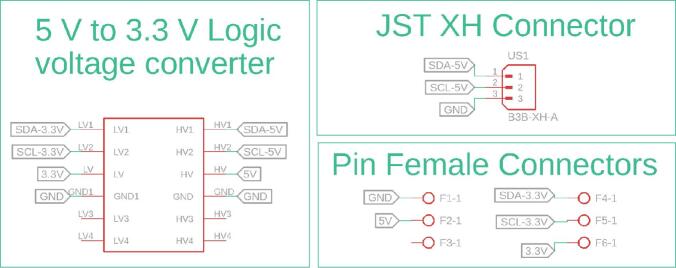
Detailed electronic schematic of the CPM module.

**Fig. 15:**
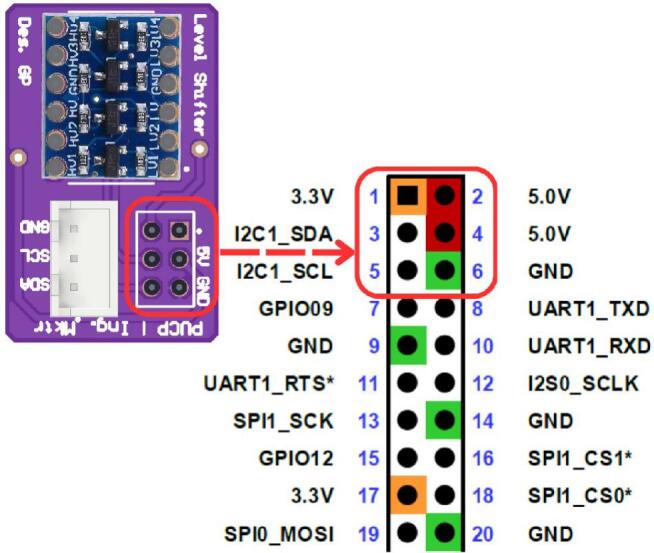
CPM PCB and GPIO connection interface on the Jetson Nano expansion header.

Fig. 14 details the internal connections of the module’s PCB. Due to the minimal interaction hardware required by the SBC, the PCB’s primary design function is to facilitate logic level conversion between the Arduino’s 5 V logic and the Jetson Nano’s 3.3 V inputs, thereby safeguarding the inputs against overvoltage damage. The board also incorporates a JST XH connector to support standard inter-module communication. Furthermore, Fig. 15 highlights the specific GPIO pinout utilized on the Jetson Nano expansion header, clarifying the physical interface amidst the high-density connector.

#### 2.4.4 Articulated Manipulation module (AMM)

**Fig. 16:**
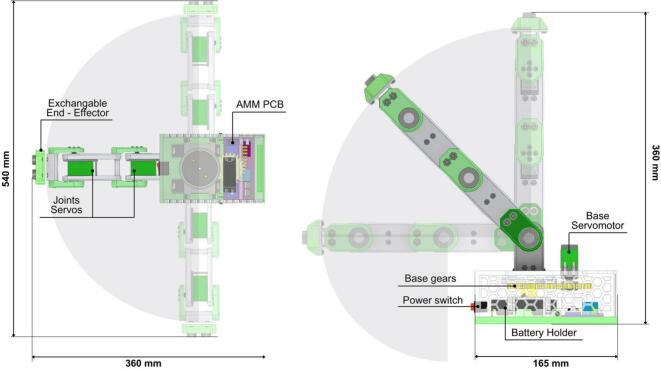
Articulated Manipulation Module dimensions and workspace.

The Articulated Manipulation Module (AMM) features an articulated robotic arm with four Degrees of Freedom (4-DOF). By default, it is equipped with an ultrasonic sensor as its end-effector; however, the mechanical interface is designed to accommodate alternative end-effectors, such as grippers, to support a broader range of tasks. Each joint of the robotic arm is actuated by a 15 kg-cm servomotor. To enhance structural stability and reduce play, each servomotor is coupled with a bearing. The angular operational range for each servomotor is 180 degrees. The overall dimensions of the AMM, its reachable workspace, and principal components are illustrated in Fig. 16.

**Fig. 17:**
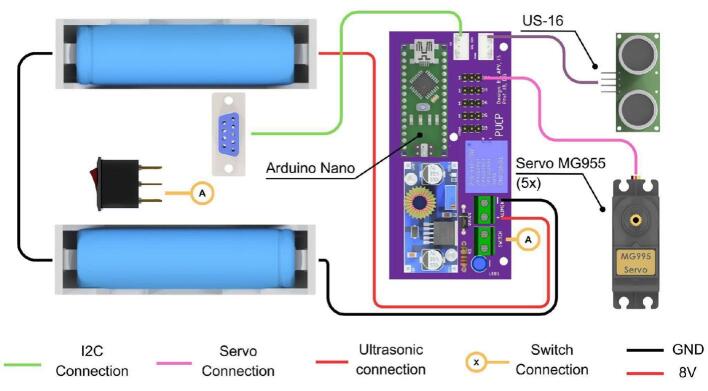
Custom designed PCB for the AMM module.

The electronic configuration of the AMM presents some distinctions compared to the other modules, primarily concerning power supply and microcontroller selection. The AMM utilizes two 18,650 lithium-ion batteries, a reduction from other modules, and employs an Arduino Nano instead of an Arduino Uno. These choices were made to minimize the overall dimensions of the module’s electronic housing. All servomotors are controlled via PWM signals generated by the Arduino Nano. Fig. 17 details the PCB layout and the electrical connections within the AMM.

**Fig. 18:**
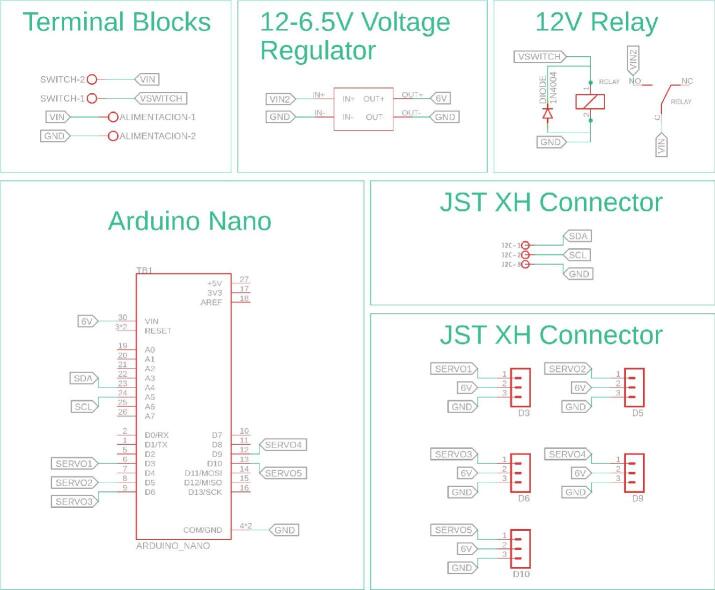
Detailed electronic schematic of the AMM module.

Fig. 18 details the internal schematic of the module’s PCB, with components organized into distinct functional groups. Key design elements include male pin headers for the five servomotors and JST XH connectors, which facilitate the standard I2C communication interface used throughout the platform.

## 3 Design files summary

All modules constituting the PlatROB platform have been designed for fabrication using Fused Deposition Modeling.

(FDM) 3D printing technology with Polylactic Acid (PLA) as the primary material. Notably, critical mechanical components such as the transmission gears and universal joints for both the ODM/DDM and ADM modules are also 3D-printed. This approach was adopted to simplify the manufacturing process and facilitate the straightforward replacement of these parts. The printing parameters for the 3D fabricated parts are shown in Table 3. Furthermore, a custom two-layer PCB has been designed for each PlatROB module to integrate its respective electronic components. Comprehensive mechanical and electronic design files for each module are provided and referenced in Table 4, Table 5, Table 6, Table 7. The tables also include part quantities and approximate printing times. Total estimated printing times are 36.8 h for the ADM, 20.3 h for the ODM/DDM, 11.7 h for the AMM, and 18.3 h for the CPM. Note that fabrication times for the PCBs are not included (N.I.) as these components were outsourced.

**Table 3:** 3D printing parameters for fabricated parts.

**Modules**	**Component Details**
Printer	Bambu Lab A1
Slicer	Bambu Studio
Material	PLA
Layer height	0.2 mm
Walls	2
Infill density	15%
Infill pattern	Grid

**Table 4:** ADM design files.

**Design filename**	**File type**	**Link**	**Qty**	**Total time**
ADM_ACKERMAN_BAR_LEFT	*.stp*	https://osf.io/ph6rk	1	∼19 min
ADM_ACKERMAN_BAR_RIGHT	*.stp*	https://osf.io/2txfn	1	∼17 min
ADM_ACKERMAN_PIECE	*.stp*	https://osf.io/7qcgx	4	∼72 min
ADM_ACKERMAN_SHAFT	*.stp*	https://osf.io/gxqhn	1	∼67 min
ADM_BASE1	*.stp*	https://osf.io/pjzd6	1	∼153 min
ADM_BASE2	*.stp*	https://osf.io/wh24m	1	∼181 min
ADM_BASE3	*.stp*	https://osf.io/cq7mj	1	∼170 min
ADM_BATTERY_TOP	*.stp*	https://osf.io/y5cmw	1	∼92 min
ADM_BEARING_HOLDER	*.stp*	https://osf.io/ypvxa	2	∼39 min
ADM_BEVEL_GEAR	*.stp*	https://osf.io/2695j	1	∼30 min
ADM_BUMPER	*.stp*	https://osf.io/q2mab	1	∼30 min
ADM_DB9_HOLDER	*.stp*	https://osf.io/fx6jz	1	∼33 min
ADM_ELECTRONIC_TOP	*.stp*	https://osf.io/fx8wp	1	∼151 min
ADM_LATERAL	*.stp*	https://osf.io/hxrm9	2	∼75 min
ADM_MAIN_SHAFT	*.stp*	https://osf.io/fmcwk	1	∼75 min
ADM_SERVO_ARM	*.stp*	https://osf.io/av526	1	∼11 min
ADM_SERVO_HOLDER	*.stp*	https://osf.io/uytfh	1	∼35 min
ADM_SHAFT	*.stp*	https://osf.io/yem2p	2	∼49 min
ADM_SILICONE	*.stp*	https://osf.io/s8hbd	1	∼19 min
ADM_TOP1	*.stp*	https://osf.io/wvu9d	1	∼270 min
ADM_TOP2	*.stp*	https://osf.io/utsyc	1	∼167 min
ADM_WHEEL	*.stp*	https://osf.io/vg5n3	4	∼472 min
ADM_PCB	*.brd*	https://osf.io/kt6e5	1	N.I.

**Table 5:** CPM design files.

**Design filename**	**File type**	**Link**	**Qty**	**Total time**
CPM_BASE	*.stp*	https://osf.io/mbzkt	1	∼533 min
CPM_BATTERY_HOLDER	*.stp*	https://osf.io/vxcfk	1	∼65 min
CPM_TOP	*.stp*	https://osf.io/sef8u	1	∼103 min
CPM_PCB	*.brd*	https://osf.io/6q9g5	1	N.I.

**Table 6:** ODM/DDM design files.

**Design filename**	**File type**	**Link**	**Qty**	**Total time**
ODM_BACK_SUPPORT_LEFT	.*stp*	https://osf.io/7y4jn	1	∼28 min
ODM_BACK_SUPPORT_RIGHT	*.stp*	https://osf.io/y8xun	1	∼28 min
ODM_BASE1	*.stp*	https://osf.io/p8r4a	1	∼204 min
ODM_BATTERY_HOLDER	*.stp*	https://osf.io/b2zn9	1	∼53 min
ODM_FRONT_CASE	*.stp*	https://osf.io/kwfm7	1	∼27 min
ODM_FRONT_ULTRA	*.stp*	https://osf.io/95xbc	1	∼14 min
ODM_FRONT_SUPPORT_LEFT	*.stp*	https://osf.io/rwp6y	1	∼27 min
ODM_FRONT_SUPPORT_RIGHT	*.stp*	https://osf.io/r7vta	1	∼27 min
ODM_LATERAL_CASE	*.stp*	https://osf.io/qksbn	2	∼182 min
ODM_ULTRA_HOLDER	*.stp*	https://osf.io/9ne3u	1	∼418 min
ODM_TOP	*.stp*	https://osf.io/p5x34	2	∼36 min
ODM_WHEEL_COUPLING	*.stp*	https://osf.io/ejd34	4	∼173 min
ODM_PCB	*.brd*	https://osf.io/3buwy	1	N.I.

**Table 7:** AMM design files.

**Design filename**	**File type**	**Link**	**Qty**	**Total time**
AM_ART	*.stp*	https://osf.io/4rdhx	3	∼67 min
AM_BASE_DISK	*.stp*	https://osf.io/a8c3s	1	∼32 min
AM_BASE_LINK_BEARING	*.stp*	https://osf.io/djbyc	3	∼93 min
AM_BASE_LINK	*.stp*	https://osf.io/s3qcy	3	∼65 min
AM_BASE	*.stp*	https://osf.io/f326j	1	∼119 min
AM_BATTERY_SUPPORT	*.stp*	https://osf.io/y362q	1	∼35 min
AM_BEARING_ART	*.stp*	https://osf.io/sygb3	3	∼70 min
AM_CASE	*.stp*	https://osf.io/cxt8w	1	∼299 min
AM_GEAR_MAIN_SHAFT	*.stp*	https://osf.io/54v3z	1	∼48 min
AM_GEAR_SERVO	*.stp*	https://osf.io/qjxpb	1	∼23 min
AM_LINK_BEARING	*.stp*	https://osf.io/mwuq8	1	∼37 min
AM_LINK	*.stp*	https://osf.io/r6kvu	1	∼48 min
AM_MAIN_SHAFT_PLATE	*.stp*	https://osf.io/fb6un	1	∼23 min
AM_TOP	*.stp*	https://osf.io/ycuj7	1	∼59 min
AM_ULTRA_SUPPORT	*.stp*	https://osf.io/32yus	1	∼45 min
AM_ULTRA_SUPPORT_V2	*.stp*	https://osf.io/a79gf	1	∼36 min
AM_PCB	*.brd*	https://osf.io/9yzh5	1	N.I.

## 4 Bill of materials summary

This section provides a comprehensive list of components required for the replication of the PlatROB kit. For clarity, the bill of materials (BOM) is organized to distinguish between different types of parts. Specifically, Table 8 enumerates the mechanical components utilized in the platform. Following this, Table 9 details the electronic components that are integrated directly onto the PCBs for each module. Finally, Table 10 lists the discrete electronic components that are assembled or connected externally to the PCBs.

**Table 8:** Bill of mechanical components.

Modules	**Designator**	**Component**	**Cost per unit** **(PEN)**	**Source of materials**	**Material type**
ADM-ODM	Bolts and Nuts	Bolts and Nuts	0,2	SAISAC Peru	Metal
ODM	MecanumWheel	Mecanum Wheel Kit 80 mm	116	DF Robot	Plastic
ADM-AMM	Servoplate	Servoplate	3	Electromania Peru	Composite
ODM	PLA	PLA esun 1 kg	60	Krear3D Peru	Plastic
ADM-AMM	Bearings	Bearings 28x12x8	2	Duccase Peru	Metal

**Table 9:** Bill of PCB components.

Modules	**Designator**	**Component**	**Cost per unit** **(PEN)**	**Source of materials**	**Material type**
ADM-ODM-AMM	Relay	Relay 12VDC 10A 250VAC	2	HIFI Peru	Composite
ADM-ODM-CPM-AMM	Protection-Diode	Diode UltraFast UF4007	0,3	HIFI Peru	Composite
ADM-ODM-CPM-AMM	Switch	KCD1-104 Switch Interruptor	1	HIFI Peru	Composite
ADM-ODM-CPM-AMM	Terminal	2 pins connection terminal 10A3	0,4	HIFI Peru	Composite
ADM-ODM-CPM	JST XH3	JST XH 3 pins	0,9	HIFI Peru	Composite
ADM-ODM	JST XH4	JST XH 4 pins	1,1	HIFI Peru	Composite
ODM-AMM	Led	Blue diffused led 5 mm	0,2	HIFI Peru	Composite
ADM-AMM	Header Female	HeaderFemaleLongx40	0,7	HIFI Peru	Composite
ADM-ODM-CPM- AMM	Header Male	HeaderMalex40	1,2	HIFI Peru	Composite
ODM	SN74LS04N	SN74LS04N Six gates NOT	1,4	HIFI Peru	Composite
ODM	Resis220	Resis 220 Ohm 1 W	0,2	HIFI Peru	Composite
CPM	Level shifter	Level shifter 3.3–5 V	2,5	Electronica Peru	Composite
ADM	ODM PCB	PCB	8,9	JLC	Composite
ODM	ODM PCB	PCB	8,9	JLC	Composite
CPM	CPM PCB	PCB	8,9	JLC	Composite
AMM	AMM PCB	PCB	8,9	JLC	Composite

**Table 10:** Bill of electronic components connected externally to the PCBs.

Modules	**Designator**	**Component**	**Cost per unit** **(PEN)**	**Source of materials**	**Material type**
ADM-ODM-CPM- AMM	Battery	Battery 18,650 1600 mAh	8,4	HIFI Peru	Composite
ODM	UltrasonicSensor	UltrasonicSensor US-016 5VDC	12,8	HIFI Peru	Composite
ADM-ODM	Arduino Cable	Arduino Cable type B	3,8	HIFI Peru	Composite
ADM	Charger	Charger de 2 Batteries 18,650	11,7	HIFI Peru	Composite
AMM	Arduino Nano	Arduino Nano	9,8	HIFI Peru	Composite
ADM-ODM-CPM- AMM	XL4005	Step down XL4005	9,8	SAISAC Peru	Composite
ODM	Charger	Charger de 2 Batteries 18,650	11,7	HIFI Peru	Composite
ADM-ODM-CPM-AMM	DB9 Female	DB9 Female	1	HIFI Peru	Composite
ADM-ODM-CPM-AMM	DB9 Male	DB9 Male	0,9	HIFI Peru	Composite
ADM-ODM-CPM	Holder x3	BatteryHolder x3	4	NAYLAMP Peru	Composite
AMM	Holder x1	BatteryHolder x1	0,2	NAYLAMP Peru	Composite
ADM-ODM	Arduino Uno	Arduino Uno R3	90	Creatividad Ahora	Composite
ODM	MotorDC 12 V	Motor JGY-370B 90RPM	68	Peru Aliexpress	Composite
ADM	Motor 37D	Motor 37D	60	NAYLAMP Peru	Composite
ODM	ODM Driver	TB6612FNG Dual Motor Driver	26,8	Pololu	Composite
ADM	ADM Driver	Carrier TB9051FTG Dual Motor Driver	162,8	Pololu	Composite
ADM	Servomotor	Carrier Yahboom 15 kg Servo	71,04	Yahboom	Composite

For a granular view of the components required for each module, Table 11 provides links to detailed specifications, costs, and specific quantities. These resources also include the list of bolts necessary for the assembly of each module.

**Table 11:** Module component details.

**Modules**	**Component Details**
Ackermann Drive Module (ADM)	https://osf.io/8fxzb
Omnidirectional Drive Module (ODM/DDM)	https://osf.io/d3utg
Control and Processing Module (CPM)	https://osf.io/a2gw5
Articulated Manipulation Module (AMM)	https://osf.io/eha6u

## 5 Build instructions

**Fig. 19:**
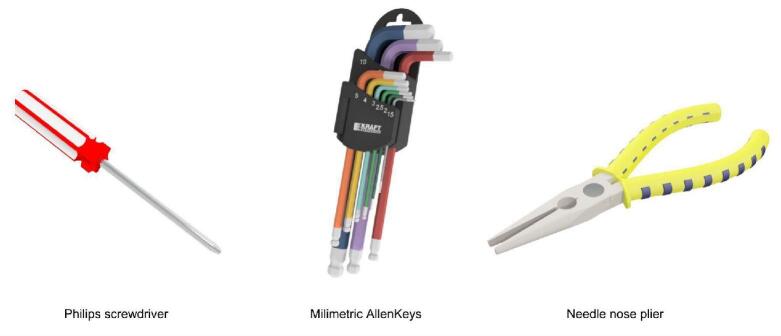
Tools required for PlatROB assembly.

To guide users through the multi-step assembly of each module within the proposed robotic platform, a detailed, step-by-step building manual has been created for each. These manuals feature comprehensive instructions and visual illustrations that cover the entire building process. All building manuals can be accessed from the OSF project repository (10.17605/osf.io/KU92V); in Fig. 19 specifies the main tools needed for the complete assembly of the educational platform. Additionally, a FDM printer and soldering iron are needed for printing 3D parts and preparing the PCBs, respectively.

The complete assembly of a PlatROB kit can be accomplished in under 3 h, assuming the PCB is pre-soldered and all 3D-printed parts are ready. This rapid assembly time enables PlatROB to serve effectively as a tool for workshops and laboratory sessions.

### 5.1 Build instructions for the Ackermann Drive module (ADM)

The assembly of the ADM involves several key sub-assemblies, detailed below.

#### 5.1.1 ADM PCB preparation

**Fig. 20:**
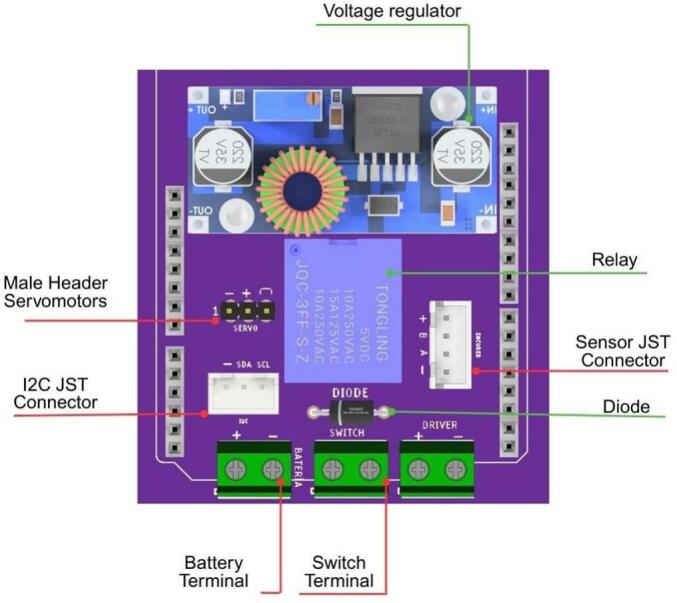
ADM-PCB: components’ positions for soldering.

The assembly begins with the soldering of the main ADM PCB as shown in Fig. 20. However, the DC motor is not mounted directly on the PCB; rather, it is connected to the VNH5019 as shown in Fig. 7.

1. The JST headers are connectors for the sensors, and the I2C extension should be soldered on the PCB.

2. The voltage regulator is soldered on the PCB using single Male Headers.

3. Solder Male Headers for the Servomotors.

4. Finally, solder the diode, the terminals, and the relay.

#### 5.1.2 Drivetrain assembly

The second step focuses on the assembly of the drivetrain.

**Fig. 21:**
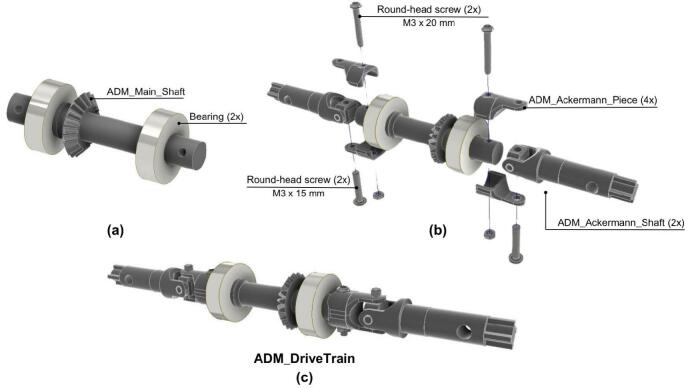
ADM: Drivetrain assembly. (a) Mounting bearings on the ADM main shaft. (b) Coupling the ADM main shaft to the ADM Ackermann shaft. (c) Fully assembled ADM drivetrain.

1. Place the two bearings at each side of the ADM_Main_Shaft as shown in Fig. 21.a.

2. The ADM_Main_Shaft is assembled to the ADM_Ackerman_Shaft using two ADM_Ackermann_Piece on each side as shown in Fig. 21.b.

3. An M3x20 round-head screw and an M3 Hexagonal nut is inserted to secure the ADM_Ackermann_Piece and the ADM_Main_Shaft.

4. An M3x15 round-head screw and an M3 Hexagonal nut is inserted to secure the ADM_Ackermann_Piece and the ADM_Ackermann_Shaft.

#### 5.1.3 Power unit

The third step details the assembly of the power unit.

**Fig. 22:**
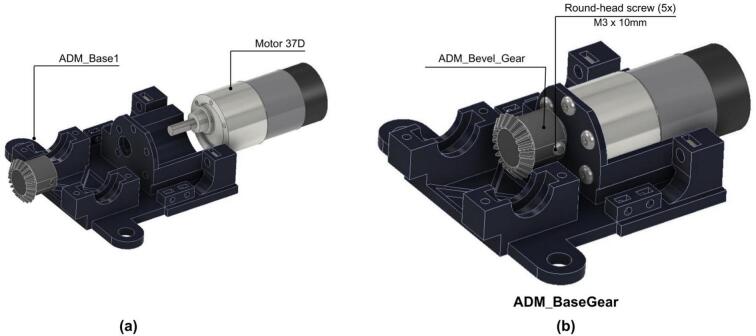
ADM: Power unit assembly. (a) Pre-assembly view of ADM Base1 and DC Motor. (b) Assembled motor and bevel gear mounted in ADM Base1.

**Fig. 23:**
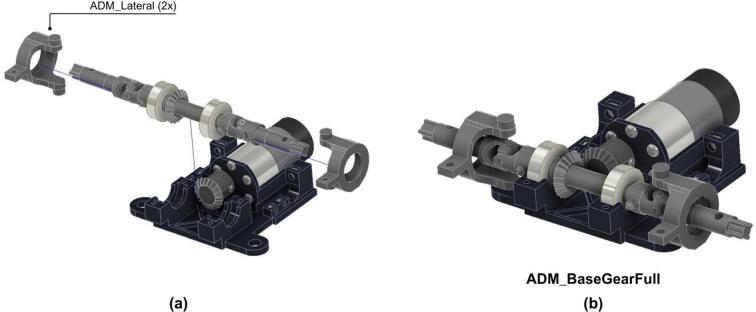
ADM: Power unit assembly with drivetrain. (a) Pre-assembly exploded view. (b) Assembled power unit showing drivetrain secured via lateral supports.

1. A DC Motor is mounted on the ADM_Base1 (Fig. 22.a).

2. The ADM_Bevel_Gear is attached to the DC Motor shaft and secured using five M3x10 round-head screws as shown in Fig. 22.b.

3. The drivetrain assembly is mounted on the ADM_Base1 and fixed using two ADM_lateral as shown in Fig. 23.

#### 5.1.4 Vehicle chassis

The fourth step explains the assembly and integration of the chassis. The rear wheel assembly is performed following the next steps.

**Fig. 24:**
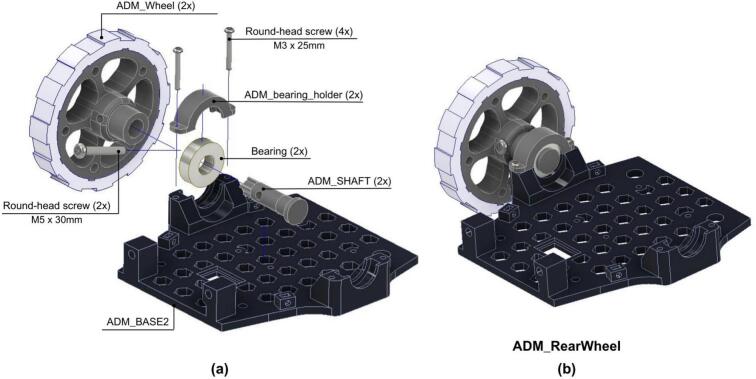
ADM: Rear wheel assembly. (a) Pre-assembly exploded view showing parts positioning. (b) Fully assembled rear wheel mounted on ADM Base2.

1. The ADM_Shaft is inserted into the ADM_Wheel locating a bearing in between.

2. Then, the ADM_Wheel with the shaft is mounted over the ADM_Base2 using the ADM_bearing_holder.

3. The items are then secured using two M3x25 round-head screws as shown in Fig. 24 and placed with the ADM_Shaft.

4. The same steps are repeated for the other rear wheel.

5. For the front wheels, the steps 1, 2 and 3 are repeated only using the ADM_Ackerman_shaft to place the wheel.

Later, the integration of the components that conform the chassis is done following the next steps.

**Fig. 25:**
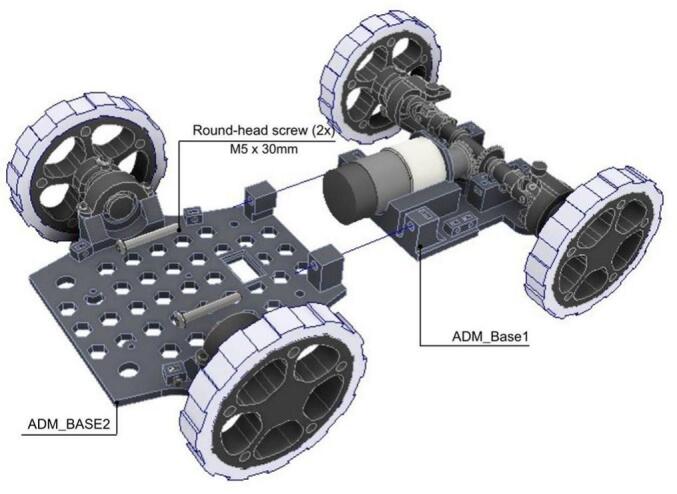
ADM: Power unit assembly with chassis.

**Fig. 26:**
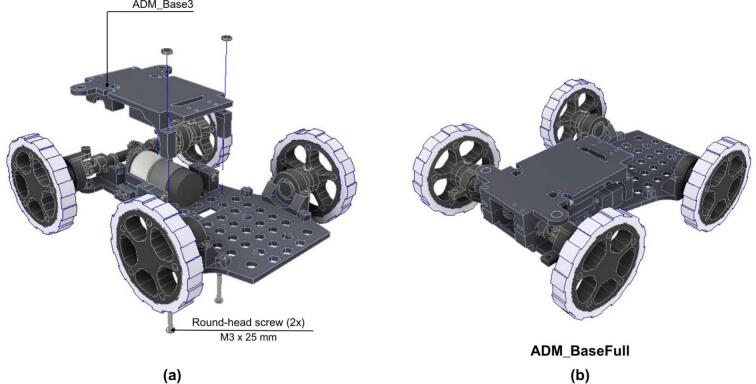
ADM: Complete chassis assembly. (a) Top cover of the ADM module base. (b) Fully assembled ADM base.

1. The ADM_Base1 is joined to the ADM_Base2 by using two M5x30 round-head screws as shown in Fig. 25.

2. The chassis is completed by placing the ADM_Base3 on top of the previous assembly.

3. The items are then secured using two M3x25 round-head screws as shown in Fig. 26.

#### 5.1.5 Electronic and holders assembly

The next step details the assembly of the electronic components.

**Fig. 27:**
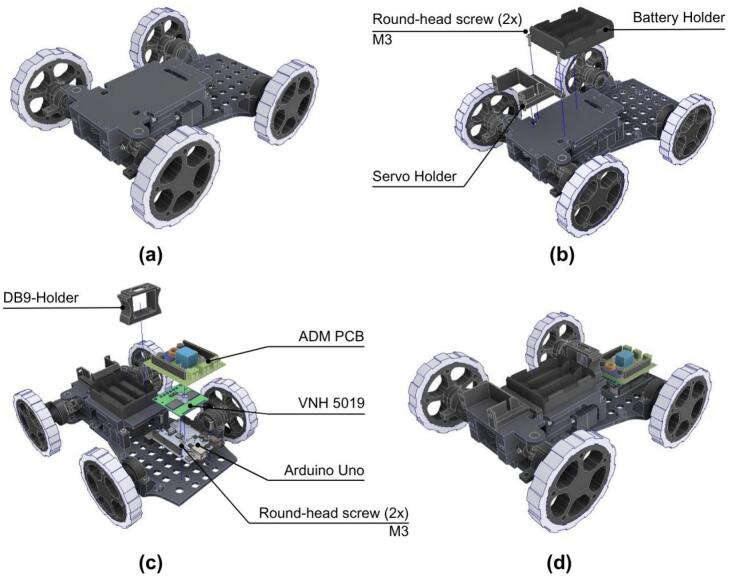
ADM: Electronics assembly. (a) Fully assembled ADM base. (b) Mounting both battery and servo holders on the ADM base. (c) Mounting Arduino Uno, ADM PCB, VHN 5019 and DB9 holder on the ADM base. (d) Fully assembled ADM electronics.

1 The Battery Holder and Servo Holder are mounted onto the fully assembled ADM base, as shown in Fig. 27.b.

2. The Arduino Uno, ADM_PCB, VHN 5019 and DB9 Holder are mounted onto the chassis, see Fig. 27.c. Only the Arduino Uno is mounted using two M3x15 round-head screws, VNH 5019 and ADM PCB are mounted above the Arduino by their pins.

#### 5.1.6 Steering mechanism

The last step details the assembly of the steering mechanism.

**Fig. 28:**
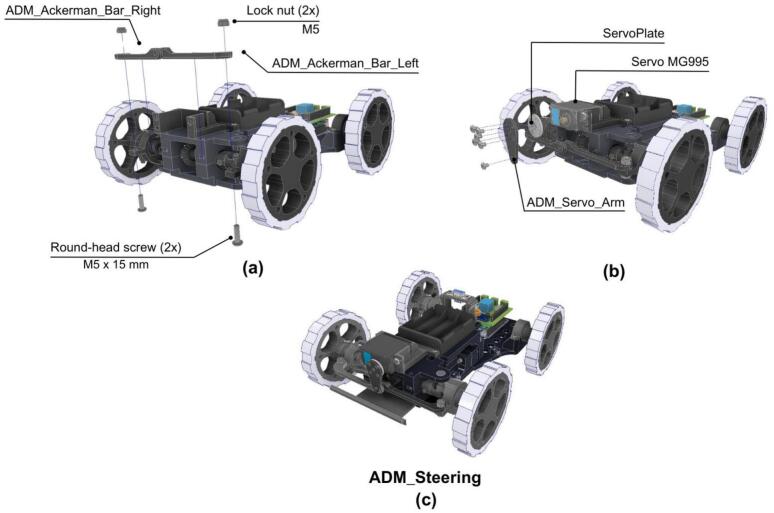
ADM: Steering mechanism assembly. (a) Steering bar connection to frame. (b) Steering servomotor coupling assembly. (c) Fully assembled steering mechanism.

1. The ADM_Ackerman_Bar_Right and ADM_Ackerman_Bar_Left are mounted onto the chassis using two M5x15 round-head screws and two lock nuts as shown in Fig. 28.a.

2. Then the Servo MG905 is mounted on the front of the chassis and joined to the ADM_Servo_Arm using the ServoPlate and using six M5x5 round-head screws, see Fig. 28.b.

#### 5.1.7 Final ADM assembly

**Fig. 29:**
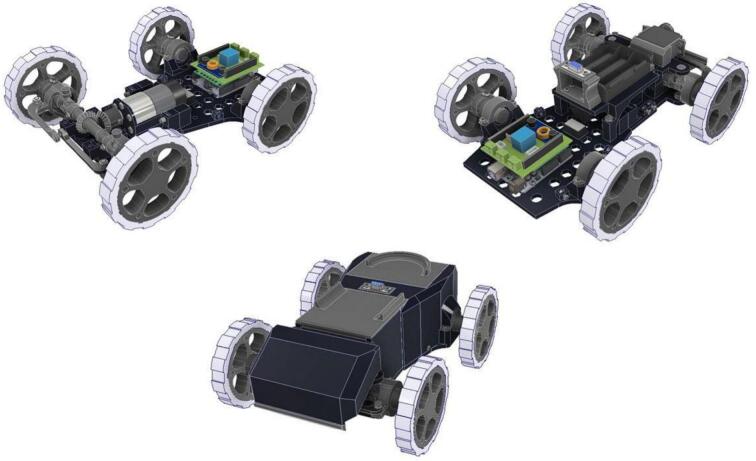
ADM: Complete module assembly.

Additional 3D-printed housing pieces are utilized to cover internal mechanical and electronic elements, protecting them from external interference. Fig. 29 illustrates the fully assembled Ackermann Drive Module.

### 5.2 Build instructions for the Omnidirectional/Differential Drive module (ODM/DDM)

The ODM/DDM assembly process consists of five stages: PCB soldering, vehicle chassis preparation, sensor integration, mounting of wheels and PCB, and final assembly with the protective cover.

#### 5.2.1 ODM/DDM PCB preparation

**Fig. 30:**
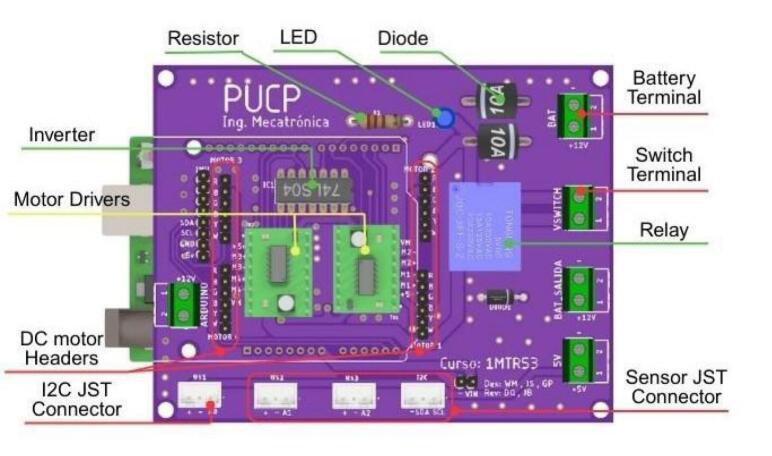
ODM/DDM-PCB: components’ positions for soldering.

The module assembly starts with soldering the main ODM/DDM PCB, which must be completed as illustrated in Fig. 30. An effective soldering sequence is to proceed from the inner components of the PCB outward, such as:

1. Solder Female Headers for the motor drivers and the inverter.

2. Solder Male Headers for the DC motors.

3. Solder JST connectors for the sensors, the I2C extension, and the terminals along the edge of the PCB.

4. Finally, solder the LED indicator, resistor, and protection diode.

#### 5.2.2 Vehicle chassis

The next stage centers on the preparation of the vehicle chassis.

**Fig. 31:**
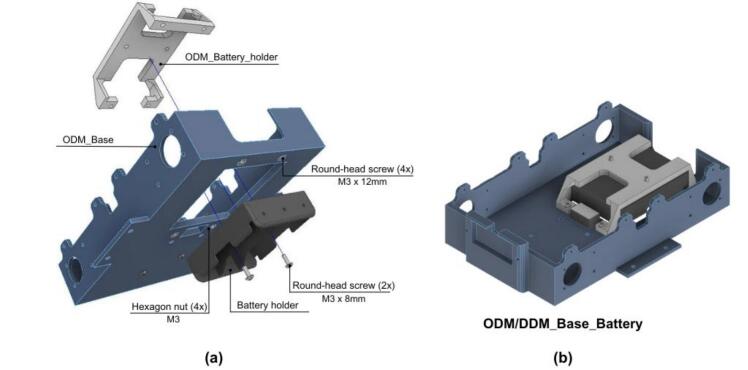
ODM/DDM: Battery holder assembly. (a) Pre-assembly exploded view. (b) Battery holder assembled to vehicle base.

**Fig. 32:**
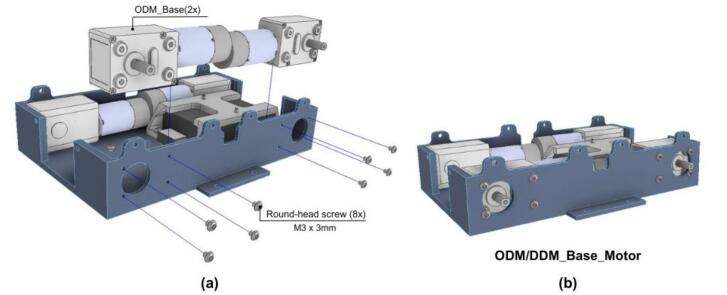
ODM/DDM: Motors assembly. a) Pre-assembly exploded view. (b) Motors mounted on ODM base.

**Fig. 33:**
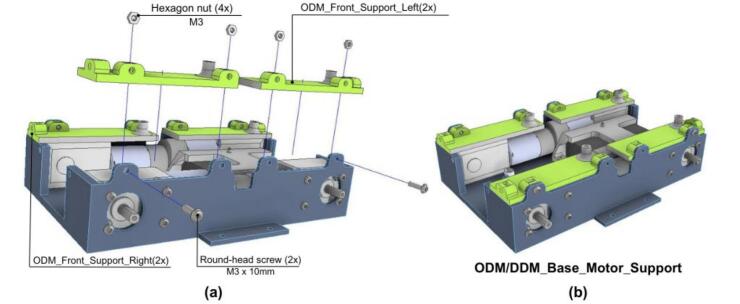
ODM/DDM: Supports assembly. (a) Exploded view showing assembly components and fasteners. (b) Fully assembled support structure.

1. Begin by placing the ODM_Battery_Holder in the ODM_Base and securing it using four hexagonal nuts as shown in Fig. 31.a, this ensures that the Battery_Holder is firmly fixed in place.

2. Next, attach the Battery_Holder to the ODM_Battery_Holder using two round-head screws; After completing this step, the assembly should resemble the one shown in Fig. 31.b.

3. Attach the four DC motors to the ODM_Base using M3x3 round-head screws, as shown in Fig. 32.a. The complete assembly should resemble Fig. 32.b.

4. Next, insert M3 Hexagonal nuts into the slots of both the ODM_Front_Support_Left and ODM_Front_Support_Right as shown in Fig. 33.a.

5. To complete this stage, fasten all support components to the ODM_Base using M3x10 round-head screws as shown in Fig. 33.b.

#### 5.2.3 Sensor integration

The third stage of the system assembly involves the modular integration of sensors.

**Fig. 34:**
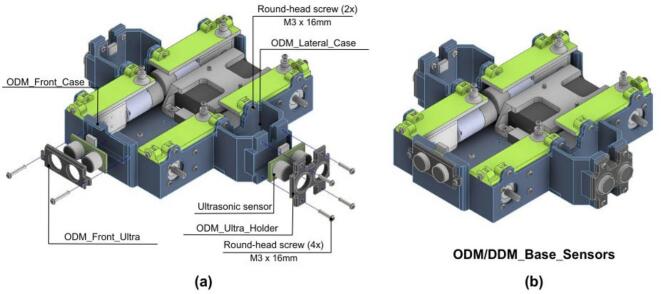
ODM/DDM: Ultrasonic sensor assembly. a) Individual components and fasteners. (b) Complete sensor installation.

1 Mount the ultrasonic sensors at the front of the chassis using the ODM_Front_Case. Secure them in place with the ODM_Front_Ultra bracket and M3x16 screws as shown in Fig. 34.a.

2. Position ultrasonic sensors at the lateral mounting points by attaching the ODM_Lateral_Case and securing it with the ODM_Ultra_Holder and M3x16 screws as shown in Fig. 34.a. The assembled vehicle at this stage should appear as shown in 34.b.

3. Ensure that all sensor wiring is properly routed and that sufficient clearance is maintained to avoid obstruction of sensor operation.

4. (Optional) Replace the ultrasonic sensors with other Time-of-Flight (ToF) sensors (e.g., infrared or laser-based units) if required by the application.

#### 5.2.4 Wheel and PCB mounting

The wheels and PCB are then mounted to the vehicle chassis.

**Fig. 35:**
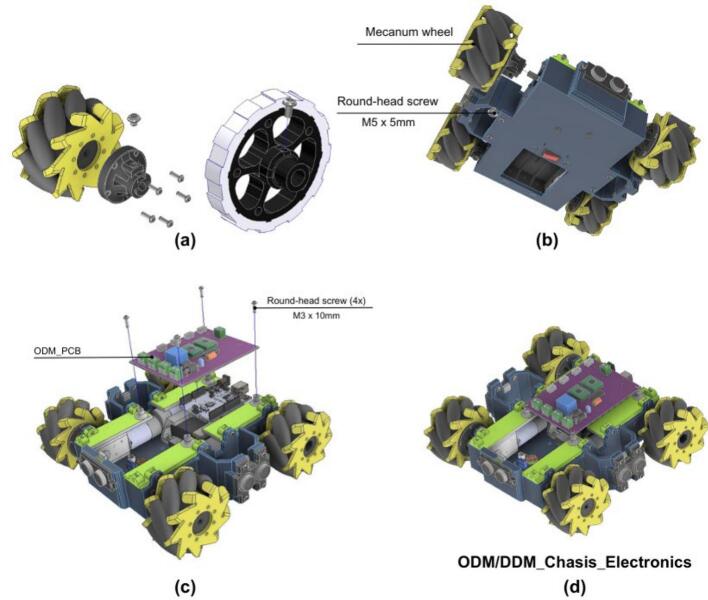
ODM/DDM: Wheel and electronics assembly. (a) Mecanum wheel hub components. (b) Wheels mounted on chassis. (c) PCB installation. (d) Complete chassis with wheels and electronics.

1. Attach the custom ODM_Wheel Coupling to the wheels using four self-tapping screws as shown in Fig. 35.a.

2. Next, secure the wheels to the motor axles using an M5x5 round-head screw as shown in Fig. 35.b.

• Use omnidirectional wheels for ODM configuration.

• Use standard wheels for differential drive (DDM) configuration.

3. Secure the ODM PCB on top of the support structures, positioned above the motors using M3x10 round-head screws, Fig. 35.c.

4. Finally, route all cables between motors, sensors, and the PCB to ensure proper electrical connectivity and prevent interference with moving components. At this stage, the vehicle should look as shown in Fig. 35.d.

#### 5.2.5 Final ODM/DDM assembly and enclosure

The last stage protects and finalizes the module.

**Fig. 36:**
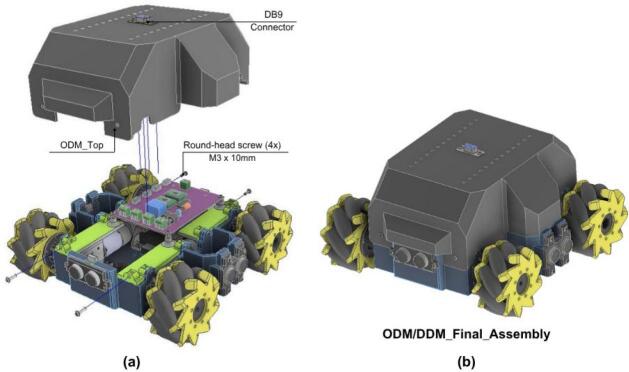
ODM/DDM: Final assembly with protective enclosure. (a) Installation of protective cover and DB9 connector. (b) Complete enclosed module.

1. Mount the DB9 connector on the cover as shown in Fig. 36.a. This allows communication and integration with other PlatROB modules.

2. Install the 3D-printed protective cover (ODM_Top) over the assembly.

3. Ensure that the cover is properly aligned with the base and support structures.

4. Verify that all parts are secured and check that the completed module matches the configuration shown in Fig. 36.

### 5.3 Building instructions for control and Processing module (CPM)

The assembly procedure for the CPM is comparatively straightforward because of its primary function of housing the SBC. The core components integrated into this module are the NVIDIA Jetson Nano SBC, the DB9 connector for inter-module communication, and the 18,650 battery holder for power supply.

**Fig. 37:**
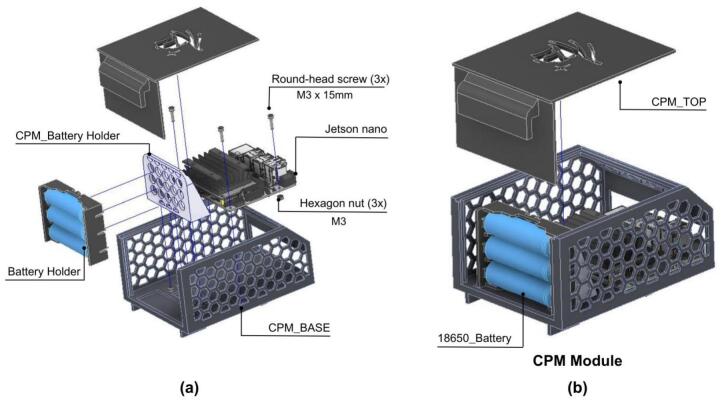
CPM: Component integration. (a) Exploded assembly view. (b) Completed CPM module with enclosed batteries.

1. Attach the CPM_Battery_Holder with the Battery_Holder as shown in 37.a.

2. Attach the CPM_Battery_Holder and Jetson Nano to the CPM_Base and secure them with round-head screws.

3. Slide the CPM_Top into the CPM_Base as shown in Fig. 37.b.

### 5.4 Building instructions for the Articulated Manipulation module (AMM)

The AMM assembly is organized into four main sub-assemblies: PCB preparation, the base unit that contains the control electronics, the articulated arm, and the complete AMM assembly.

#### 5.4.1 AMM PCB preparation

**Fig. 38:**
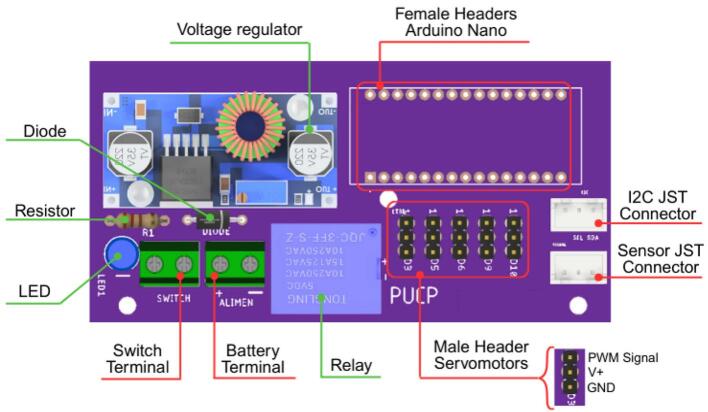
AMM-PCB: Components’ positions for soldering.

Before starting the AMM assembly process, PCB soldering should be completed in the position shown in Fig. 38. A suggested soldering order is presented, and the cable connection is also performed during other steps.

1. The Female Headers, terminals, and JST connectors should be soldered on the PCB.

2. Then, the LED indicator, resistor, and protection diode should be soldered.

3. The voltage regulator is soldered on the PCB using single Male Headers.

4. Finally, the Arduino Nano is connected to the Female Headers. As a reference, the USB connector of the Arduino Nano must face the edge of the PCB.

#### 5.4.2 Electronics base unit

This assembly stage focuses on the base unit.

**Fig. 39:**
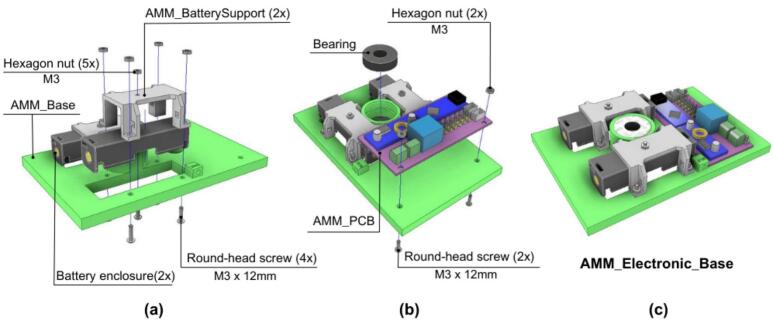
AMM: Electronics base assembly. (a) Battery enclosure and support installation. (b) Bearing and PCB integration. (c) Completed electronics base.

1. The Battery enclosure is first attached to the AMM_BatterySupport component.

2. The Battery enclosure and the AMM_BatterySupport are then secured to the AM_Base using bolts, as shown in Fig. 39.a.

3. Subsequently, a bearing is press-fitted into the central aperture of the AM_Base, as illustrated in Fig. 39.b.

4. To complete this stage, the AMM_PCB is mounted onto the AMM_Base using M3 round-head screws. Also, it is recom- mended to connect the battery cables to the corresponding terminal. The assembled electronics base is shown in Fig. 39.c.

#### 5.4.3 Articulated arm

##### 5.4.3.1 Arm base-link assembly

**Fig. 40:**
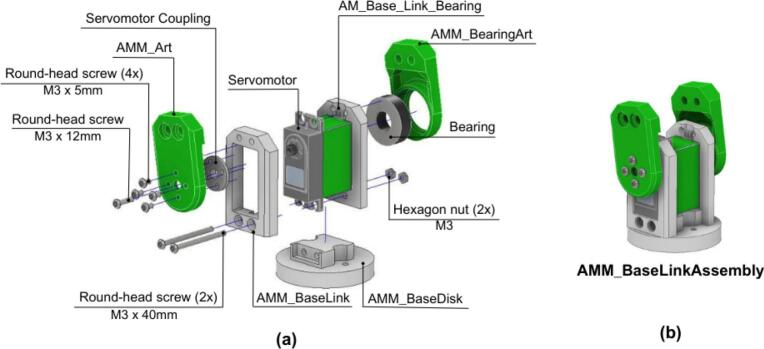
AMM: Arm base-link assembly. (a) Exploded view showing components and fasteners. (b) Assembled AMM base link.

The construction of the arm base-link is detailed in Fig. 40.

**Fig. 41:**
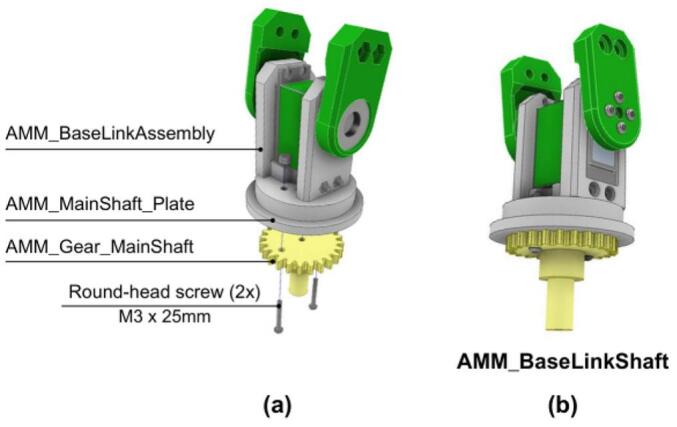
AMM: Arm base-link connection to main shaft. (a) Side view showing assembly components and mounting hardware. (b) Bottom view of assembled connection.

1. The AMM_BaseLink and AMM_Base_Link_Bearing components are fitted around the first servomotor.

2. The three parts are secured together and positioned on the AMM_BaseDisk using two M3x40 round-head screws that pass through all aligned holes.

3. Subsequently, a bearing is inserted into the designated hole of the AMM_BearingArt component, which is then mounted onto the AMM_Base_Link_Bearing.

4. The servomotor’s output shaft is then coupled to the AMM_Art component. These parts, along with the Servomotor Coupling, are joined by five M3 round-head screws, which also ensure effective torque transmission.

5. Prior to assembling the subsequent arm links, this completed AMM_BaseLinkAssembly is connected to the AMM_MainShaft_Plate and the AMM_Gear_MainShaft, as illustrated in Fig. 41.

##### 5.4.3.2 Arm links assembly

**Fig. 42:**
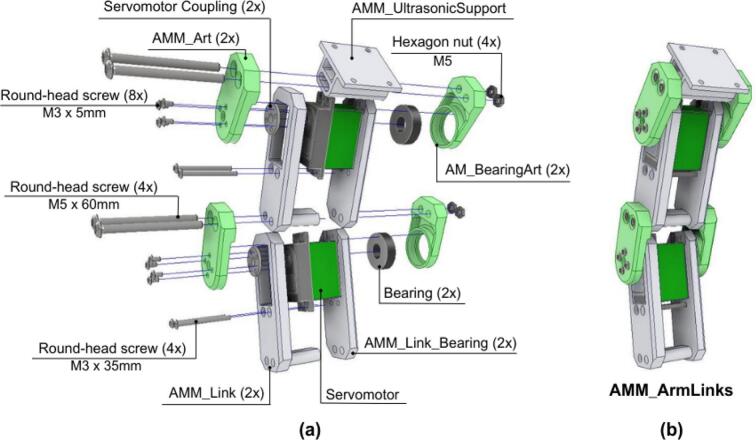
AMM: Arm links assembly. (a) Exploded view showing link components and fasteners. (b) Fully assembled arm links.

The subsequent arm links are assembled using a methodology similar to that of the base link, as shown in Fig. 42.

1. For each link, a servomotor is positioned between the AMM_Link component and the AMM_Link Bearing component. These are secured together using M3 round-head screws, see Fig. 42.a.

2. A Servomotor Coupling and a Bearing are mounted within each link assembly, following the same procedure described for the AMM_BaseLink, see also Fig. 42.a.

3. The individual arm links are then serially connected using two M5 round-head screws that pass through the four corresponding 3D-printed parts of the adjoining links, as depicted in Fig. 42.b.

4. The end-effector support (AMM_UltrasonicSupport) is assembled onto the final arm link using the same type of M5 screws, see Fig. 42.b.

##### 5.4.3.3 End-effector mounting

**Fig. 43:**
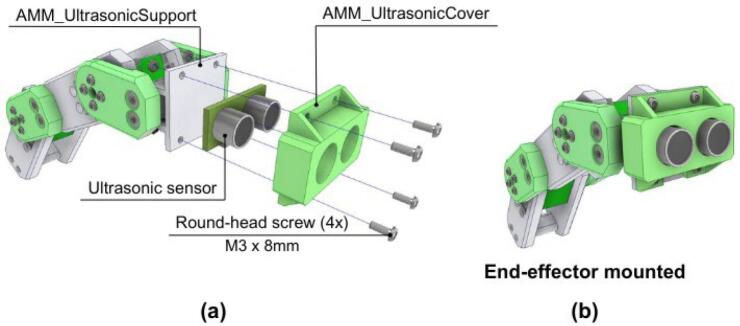
AMM: End-effector assembly. (a) Exploded view showing ultrasonic sensor mounting. (b) Completed end-effector installation.

As an example of end-effector integration, the ultrasonic sensor is attached to the AMM_UltrasonicSupport and locked in place with the AMM_UltrasonicCover using four M3 round-head screws, as shown in Fig. 43.

##### 5.4.3.4 Articulated arm assembly

**Fig. 44:**
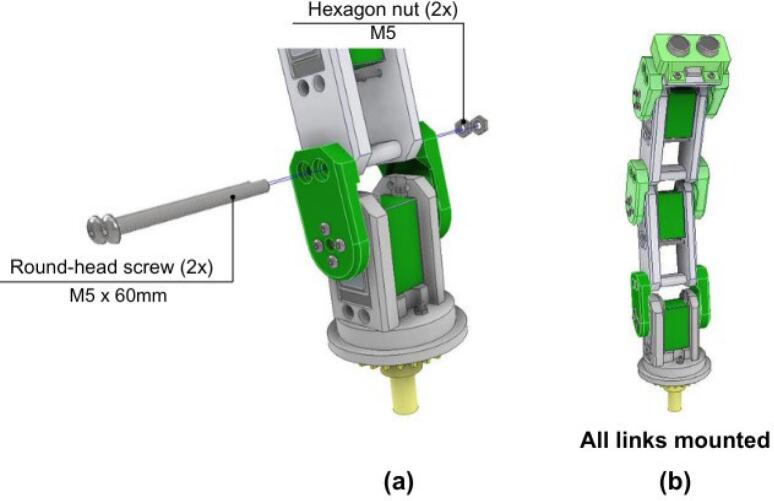
AMM: Articulated arm assembly. (a) Base-link connection to arm links. (b) Fully assembled arm structure.

To form the complete arm structure, the AMM_BaseLinkShaft assembly is connected to the chain of AMM_ArmLinks using two M5 round-head screws, as illustrated in Fig. 44.

#### 5.4.4 Final AMM assembly: Integrating the electronics base unit and articulated arm

The final stage involves integrating the assembled articulated arm structure with the electronics base.

**Fig. 45:**
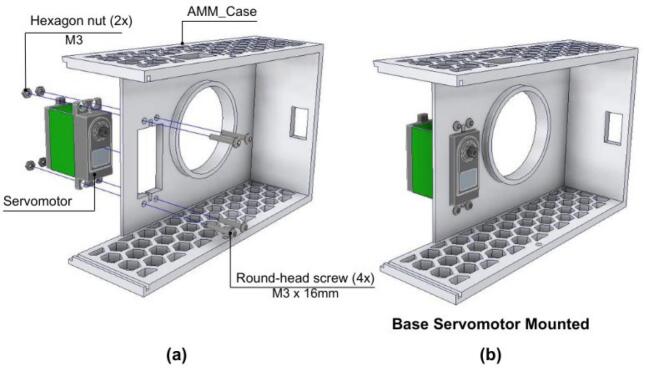
AMM: Base servomotor assembly. (a) Servomotor installation with mounting hardware. (b) Completed servomotor installation.

**Fig. 46:**
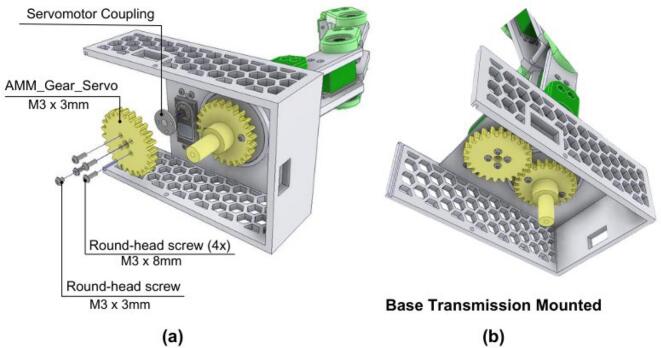
AMM: Base transmission assembly. (a) Gear train components with mounting hardware. (b) Fully assembled base transmission system.

**Fig. 47:**
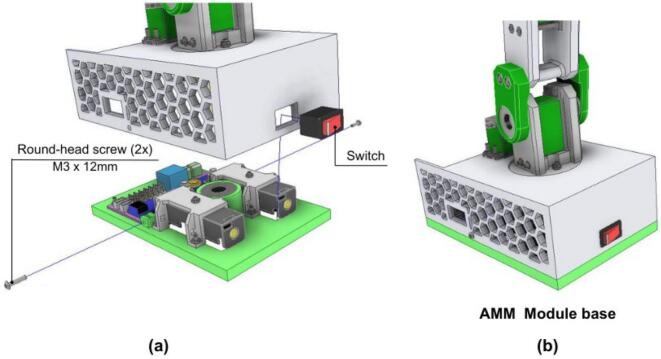
AMM: Final assembly. (a) Electronics base with case installation. (b) Complete AMM module.

1. A servomotor (for base rotation) is placed into the AMM_Case and secured with four M3 round-head screws, as shown in Fig. 45.

2. The Servomotor Coupling component and the AMM_Gear_Servo are then attached to this servomotor’s shaft using round-head screws, as depicted in Fig. 46.

3. The previously assembled complete articulated arm is then inserted into the central aperture of the AMM_Case. During this step, it is crucial to ensure proper alignment of the gears, as indicated in Fig. 46.

4. The power-on switch is installed and its cable should be connected to the PCB, as shown in Fig. 47.

5. The AMM_Case (containing the articulated arm and base servomotor) is also to be connected to the AMM_Base (housing the electronics). Care must be taken to align the bearing in the AMM_Base with the main shaft extending from the AMM_Case assembly.

6. Finally, the servomotor connectors and sensor should be connected into the PCB. The completed AMM is shown in Fig. 47.

## 6 Operation instructions

### 6.1 Programming validation code

The PlatROB modules are designed primarily to be programmed using the Arduino Integrated Development Environment (IDE). This environment was selected for its ease of use, extensive online documentation, and readily available libraries to interface with various sensors and actuators.

To facilitate initial setup and ensure correct functionality post-assembly, a set of validation codes is provided. These codes, summarized in Table 12, are designed to test the core features of each module.

**Table 12:** Source code for module validation.

**Modules**	**Component Details**
ADM Servo Validation code (.ino)	https://osf.io/4rws6
ADM Motor Validation code (.ino)	https://osf.io/s3dmy
ODM/DDM Validation code (.ino)	https://osf.io/r4wba
CPM Validation code master (.py)	https://osf.io/8qehv
CPM Validation code slave (.ino)	https://osf.io/qp5bh
AMM Validation code (.ino)	https://osf.io/nx3g9

**Fig. 48:**
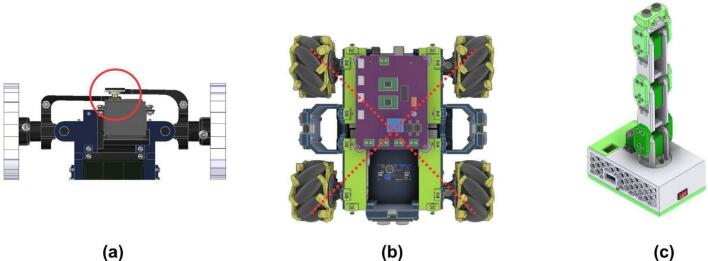
Module assembly verification views. (a) ADM motor and steering alignment check (90° orientation). (b) ODM wheel configuration (bottom view). (c) AMM module default position.

**Ackermann Drive Module (ADM) validation** The ADM incorporates a 996R servomotor for steering control and a DC motor for wheel propulsion. Critical to ADM validation is the proper mechanical alignment of the steering servomotor, as depicted in Fig. 48.a. A correct alignment ensures that when the servomotor is commanded to its neutral position (typically 90°), the vehicle is oriented for straightforward motion. Table 12 provides links to specific Arduino codes: one for calibrating the servomotor’s neutral position and another for testing the drivetrain’s operational integrity.

**Omnidirectional/Differential Drive Module (ODM/DDM) validation** Validation of the ODM/DDM focuses on confirming the operational status of its sensors, motors, and motor drivers. A key mechanical check involves verifying the correct orientation of the omnidirectional wheel rollers, which should form an 'X’ pattern when viewed from above, as illustrated in Fig. 48.b. This configuration is essential for achieving true omnidirectional movement. The validation code for the ODM/DDM, listed in Table 12, facilitates testing of all integrated sensors and actuators. Running the code will perform two actions: first, each motor independently rotates forward and backward to verify correct wiring and functionality. Second, the readings from the ultrasonic and IMU sensors are output to the console at a rate of 1 Hz.

**Control and Processing Module (CPM) validation** The CPM communicates with other modules via the I2C protocol. Validation, therefore, centers on verifying the integrity of this I2C communication link. Specific validation scripts are provided: a Python script for the NVIDIA Jetson (master) and an Arduino sketch for any connected slave module (e.g., ADM, ODM/DDM, AMM). During the test, the NVIDIA Jetson transmits a predefined character (e.g.,’A’) over I2C. The receiving Arduino module is programmed to acknowledge this transmission, send a response, and activate an indicator LED to confirm successful bidirectional communication.

**Articulated Manipulation Module (AMM) validation** Validation of the AMM necessitates a homing procedure for its servomotors to establish a known reference position, ensuring accurate angular control of the arm’s joints. The defined home position, illustrated in Fig. 48.c. typically involves setting each servomotor to a 90-degree angle. The validation code for the AMM, listed in Table 12, not only commands the arm to this initial pose but also verifies the functionality of the integrated ultrasonic sensor (if equipped as the end-effector).

### 6.2 Operating instructions for the mobile robot

This subsection details the procedures for operating fully assembled mobile robot configurations using the PlatROB modules. These instructions cover common operational scenarios beyond initial validation, such as teleoperation and basic autonomous functionalities.

**Prerequisites:**

- Ensure all relevant modules (e.g., ADM or ODM/DDM, CPM, power sources) are correctly assembled and have passed individual validation tests as described in Section 6.1.
- Verify all inter-module connections (power and data, e.g., I2C via DB9 connectors) are secure.
- Ensure a computer with keyboard input is connected to the same network as the CPM for Wi-Fi based teleoperation.

**Teleoperation Setup and Execution:**

- Prepare the Arduino code by uploading the corresponding teleoperation program (ADM/ODM_Teleoperation.ino) to the robotic vehicle using Arduino IDE.
- In the CPM, open the corresponding server program (ADM/ODM_Server.py) in any Python IDE and modify the corresponding port to ensure correct communication.
- In an external computer, open the corresponding client program in any Python IDE and modify the server IP to correspond to the CPM.
- Execute the server program on the CPM.
- Execute the client program in an external computer and enter teleoperation commands (’W’,’A’,’S’,’D’,’Q’,’E’,’X’).
- The code will send this command to the CPM via sockets or MQTT and perform the corresponding action on the vehicle.

### 6.3 Operating instructions for the AMM module

This subsection presents the instructions for operating the AMM module. The objective is to send joint angles to the module. These instructions assume the AMM has passed initial validation.

**Prerequisites:**

- Ensure the AMM is correctly assembled and validated.
- Verify power supply and communication interface (e.g., direct USB connection to a computer running Arduino IDE, or I2C connection if controlled via CPM). It is suggested to start with the USB connection.
- Download the program AMM Commands Receiver.ino and open it with the Arduino IDE.

**Communicating with the AMM module:** This procedure outlines how to control the AMM module by sending commands from a Python script to the Arduino Nano via a USB connection.

- Prepare the Arduino Nano code by uploading the program called AMM_Commands_Receiver.ino.
- In any Python IDE, open the file AMM_Teleoperated_Commands.py and modify the port to ensure correct communication.
- Execute the Python code and be ready to introduce the 4 AMM joint angles as a string (e.g. 45, 30, 60, 50).
- The code will convert these angles to a command string including a header and checksum to validate the message completeness.

Table 13 describes the necessary software resources required to execute the operating procedures described above. It provides direct links to the Arduino firmware and Python scripts for teleoperating the mobile platforms and controlling the AMM.

**Table 13:** Source code for operating the ADM/ODM and AMM modules.

**Modules**	**Component Details**
ADM_Teleoperation (.ino)	https://osf.io/xmf6u
ADM_Server (.ino)	https://osf.io/9nsrc
AMM_Commands_Receiver (.ino)	https://osf.io/49sf5
AMM_Teleoperated_Commands (.py)	https://osf.io/k2aeg

## 7 Validation and characterization

### 7.1 ADM module

#### 7.1.1 ADM maximum payload

Load tests on the ADM module determined its maximum capacity. The procedure involved attaching a hopper to the chassis, adding weight in 1 kg increments, and driving the vehicle through a circuit with straight and curved sections. The module achieved a maximum load capacity of 10 kg. The front wheel axles were identified as the critical stress point, as they failed first when attempting directional changes under heavy loads. While performance was optimal up to 10 kg, a load of 11 kg caused excessive wear to the cardan-type joints.

#### 7.1.2 ADM turning radius

One of the key performance metrics for the ADM module was its steering capability, which was essential for overcoming the sharp turns of the test circuit. Initial iterations exhibited unsatisfactory results, primarily due to wheel slippage and constraints in the turning capacity of the joints. The slippage issue was mitigated by integrating rough silicone wheels, while the turning radius was enhanced through optimization of the transmission system. To validate this improvement, the vehicle’s turning radius was measured by having it pivot on its own axis, yielding a result of approximately 25 cm.

#### 7.1.3 ADM battery autonomy

To determine the maximum operational duration for planning student activities, the module underwent continuous motion testing. Simulating a full hardware configuration with a 3 kg payload, the vehicle operated for an average of 120 min on a 2800 mAh lithium-ion battery.

### 7.2 CPM module

#### 7.2.1 CPM battery autonomy

The operational autonomy of the CPM module is contingent upon the NVIDIA Jetson Nano’s computational load. When powered by 2800 mAh lithium-ion batteries, the system operates for approximately 100 min during low-intensity tasks, such as teleoperation via TCP or MQTT. This duration is substantially reduced to a 40–60 min range when executing demanding neural network computations.

### 7.3 AMM module

#### 7.3.1 AMM battery autonomy

To determine the maximum operational duration for planning student activities, the module underwent continuous motion testing. In those tests, the arm carried 100gr of payload in the last joint (joint 4) simulating a normal use in class activities, the vehicle operated for an average of 100 min on a two 2800 mAh lithium-ion battery.

#### 7.3.2 AMM maximum payload

Based on motor calculations, the robotic arm’s estimated payload is 450 g. This capacity is deemed appropriate for its designated function in educational settings, which involves holding sensors and reference objects for various experiments.

#### 7.3.3 AMM performance characterization

**Fig. 49:**
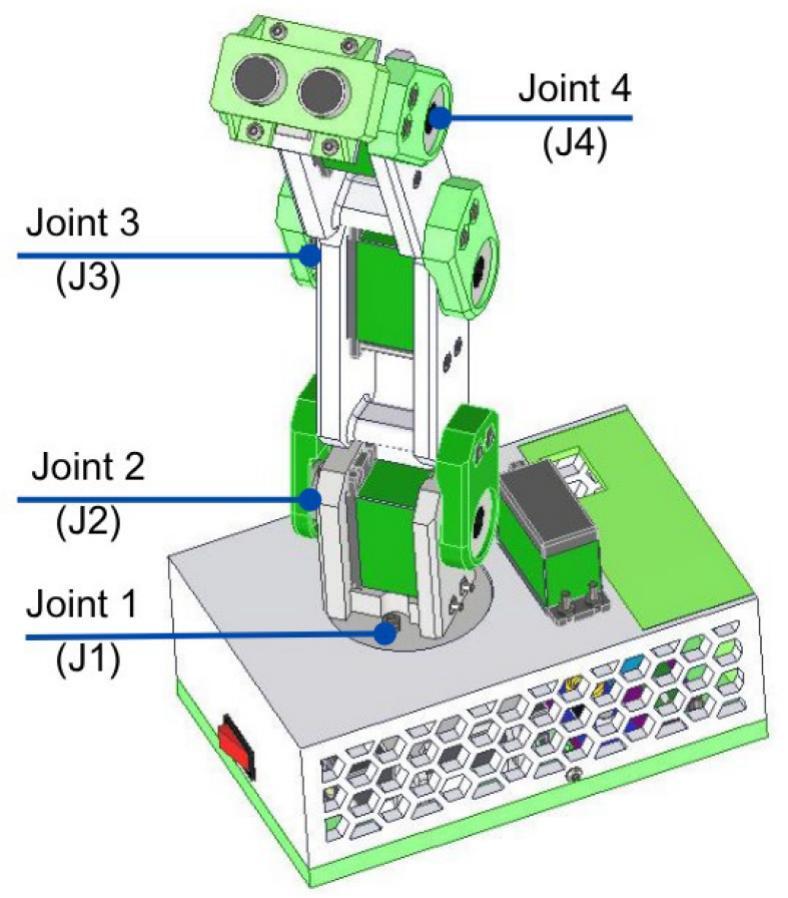
AMM joint labels used in performance characterization.

For performance characterization, payload and angular position error tests were conducted; the AMM joints used in these tests are identified in Fig. 49. Several rollover tests were conducted for each AMM joint under payloads of 150, 300 and 450 g, and operating speeds of 25%, 50%, 75%, and 100% (approximately 226^。^/s at 100%). All experiments were performed without additional mechanical fixation or clamping to the workbench, reflecting typical classroom use.

**Joint 1** (yaw-type rotation) did not exhibit rollover under any tested condition. This behavior is attributable to the payload not generating a destabilizing moment about the yaw axis, thereby maintaining dynamic stability throughout operation. **Joint 2** (first pitch axis) displayed a clear reduction in rollover angle as both payload and speed increased. With a 150 g payload, rollover occurred at approximately 60° (25% speed), 55° (50%), 40° (75%), and 35° (100%). At 300 g, these angles decreased to 40° (25%), 25° (50%), 20° (75%), and 15° (100%). At 450 g, rollover was observed at 15° (25%), 10° (50%), and 5° for both 75% and 100%. These results demonstrate that higher load and speed substantially reduce the joint’s stability margin, making rollover more likely at smaller deflection angles. **Joint 3** (second pitch axis) also exhibited sensitivity to payload and speed, though with different thresholds. At 150 g, no rollover occurred at any speed. At 300 g, rollover appeared near 90° (75% speed) and 75° (100%), with slight oscillation at 75% that did not progress to complete overturning. At 450 g, rollover was observed near 90° (25%, with oscillation), 55° (75%), and 25° (100%). This pattern corroborates the destabilizing effect of increased payload and speed observed in Joint 2, while indicating that Joint 3 maintains greater tolerance at lower speeds for equivalent loads. **Joint 4** successfully passed all test conditions without exhibiting rollover or oscillatory behavior, demonstrating robust stability across the examined operating envelope.

Overall, yaw-type joints (e.g., Joint 1) demonstrated greater inherent stability than pitch-type joints (e.g., Joints 2 and 3), which became increasingly susceptible to rollover as payload and speed increased. The systematic decrease in rollover angle for pitch joints indicates a narrowing stability margin that must be considered when defining safe operating limits.

Additional observations were made when the arm’s base was mechanically secured using a clamp or rigid attachment. Under these conditions, overall stability improved markedly. Joint 2, the most rollover-prone in free-standing tests, sustained payloads up to 600 g without overturning. This confirms that straightforward mechanical stabilization strategies can significantly extend the AMM’s operational limits, providing a practical means to accommodate heavier end-effectors or more aggressive motion profiles when required.

### 7.4 Educational assessment

The educational efficacy and impact of the PlatROB platform were systematically evaluated through its integration into a series of workshops and established undergraduate mechatronics engineering courses. This evaluation process was conducted in three distinct stages:

• **Workshops:** An initial evaluation of PlatROB ADM and CPM modules was conducted within a 6-hour workshop format, engaging mechatronics engineering students from various academic levels [24].

• **Summer Course:** A second stage consisted of applying the ADM and CPM modules in a two-month intensive summer course centered on autonomous vehicle development.

• **Full Semester Course:** The last validation phase involved the implementation of PlatROB ODM/DDM and AMM within a semester-long robotics and AI course, culminating in a final path-planning project.

**Fig. 50:**
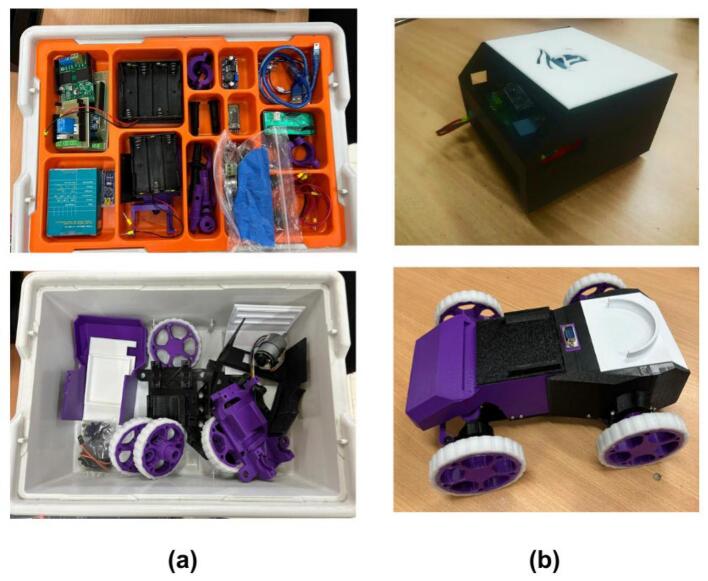
Materials for PlatROB evaluation: (a) Disassembled PlatROB modules. (b) ADM and CPM modules assembled by students.

Fig. 50 displays a representative selection of the materials provided to students, showing examples of disassembled PlatROB components alongside the fully assembled CPM module and the ADM vehicle used for coursework.

#### 7.4.1 Workshops: initial platform evaluation

The initial evaluation of engagement and learning was conducted via structured workshops with mechatronics engineering students across various academic levels. A selection criterion was established to exclude students with prior experience in comprehensive robotic projects. The study employed a mixed-method approach: quantitative pre- and post-test questionnaires measured the understanding of robotics concepts, while qualitative observational notes tracked student progress and inquiry types [24].

The workshop structure was refined based on a preceding pilot study, which highlighted the need for increased task time and the inclusion of a 20-minute theoretical introduction to the ADM and CPM modules. The final time allocations for both the pilot and the main workshop are detailed in Table 14.

**Table 14:** Pilot study and workshop duration and time allocations.

**Activity**	**Individual pre-test**	**Theoretical concepts**	**Practical assembly**	**Code integration and testing**	**Break**	**Individual post-test**	**Satisfaction survey**
Pilot Study (3 h)	15 min	0 min	60 min	75 min	0 min	15 min	15 min
Workshop (6 h)	15 min	20 min	160 min	105 min	30 min	15 min	15 min

**Fig. 51:**
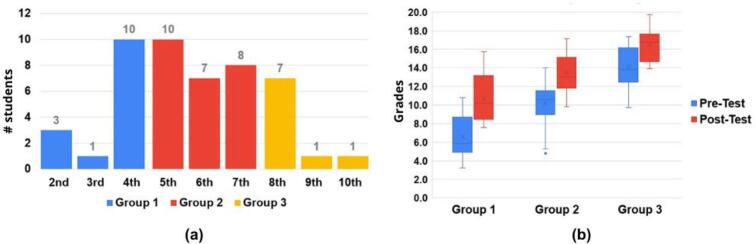
Workshop participant analysis. (a) Student distribution by academic level and experimental group. (b) Pre- and post-workshop assessment scores by group.

Data from 48 students were included in the final analysis, categorized by academic semester as shown in Fig. 51.a. Each group received instructor support during assembly and testing. Statistical analysis revealed a significant improvement (p < 0.04) in knowledge acquisition across all groups (Fig. 51.b), validating the platform's educational utility. Additionally, the average assembly time for the ADM and CPM modules was recorded at 130 ± 20.3 min (SD = 42.2; CI = 95%).

#### 7.4.2 Summer course: Application in autonomous navigation

The ADM and CPM modules were integrated into an established elective summer course on self-driving car technology. Since 2019, this course has tasked students with developing a lane-detecting vehicle using computer vision. Previous iterations relied on modified RC car frames and custom electronics [25], [26], a time-consuming approach that often limited the focus on algorithms. PlatROB was introduced to streamline hardware setup, allowing students to prioritize algorithmic development.

**Fig. 52:**
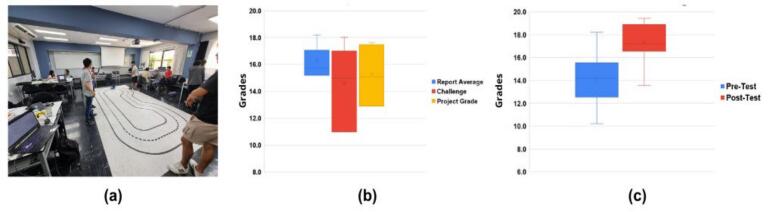
Summer 2024 course results. (a) Mobile robot racing track. (b) Student performance across assessment components: report average, challenge completion, and project grade. (c) Knowledge assessment improvement (pre- vs. post-assessments).

In the Summer 2024 session, 21 students organized into four teams used PlatROB for their capstone project: implementing an AI-based vision algorithm for autonomous lane following (Fig. 52.a). The project spanned seven laboratory sessions. During the first six guided sessions, students assembled modules, integrated a custom 3D-printed webcam mount, and collected training datasets via teleoperation. The final session featured a challenge requiring the vehicle to autonomously complete three laps.

Student performance was evaluated based on laboratory reports (40%) and the final navigation challenge (60%), scored on precision, speed, and successful turns. As shown in Fig. 52.b, all teams successfully completed the challenge (mean score x = 15.29, SD = 2.01). Furthermore, pre- and post-test questionnaires (Fig. 52.c) revealed a statistically significant improvement (p < 0.05) in conceptual understanding, with scores rising from x = 14.15 (SD = 2.05) to x = 17.35 (SD = 1.58).

#### 7.4.3 Semester-long robotics and AI course: Kinematics, path planning and system integration

The PlatROB AMM and ODM/DDM modules were integrated into “Robotics and AI,” a mandatory 6th-semester course covering mobile robotics, manipulators, and algorithms. The AMM was utilized in two practical sessions focused on teleoperation and manipulator kinematics (forward and inverse).

**Fig. 53:**
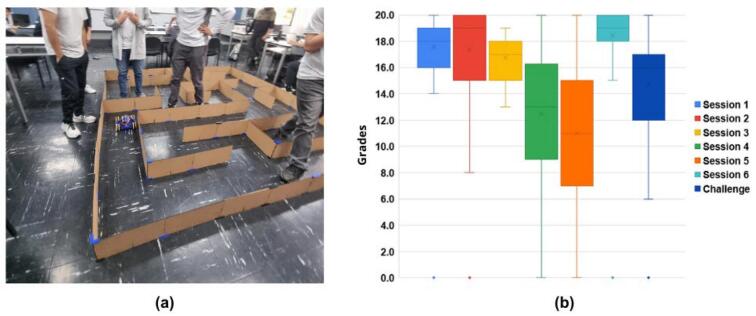
Semester-long Robotics and AI course performance. (a) Students working on final challenge with cardboard track setup. (b) Grade distribution across six weekly lab sessions and final challenge assessment.

The ODM/DDM served as the primary hardware for a semester-long project involving 98 students organized into groups of five. The project required implementing path planning algorithms (e.g., A*, Dijkstra) to navigate a predefined maze (Fig. 53.a) and developing robust path-following strategies using sensor fusion (e.g., Kalman filtering) to integrate encoder, IMU, and ultrasonic data.

Evaluation consisted of lab reports and a final navigation challenge scored on precision, speed, and collision avoidance. As shown in Fig. 53.b, student performance was generally strong across the semester. Although report scores dipped in sessions 4 and 5 due to incomplete documentation on platform integration, the final challenge yielded positive results, with a mean score of x = 14.69 (SD = 3.81) and most teams successfully meeting the criteria.

### 7.5 Conclusions

PlatROB is an open-source, cost-effective, and modular educational platform designed to enhance the teaching of mobile robotics and artificial intelligence. Its reconfigurable architecture, built with 3D-printable components and widely available electronics, addresses the challenge of limited access to affordable, versatile hardware in educational settings. The modular design, incorporating Ackermann, omnidirectional/differential drive, processing, and articulated manipulation modules, enables students to explore complex robotic systems and system integration principles progressively. Extensive use in workshops and undergraduate courses has demonstrated PlatROB’s effectiveness in improving students’ conceptual understanding and practical skills, supporting diverse learning objectives, from basic mechanics to AI-driven autonomous navigation. By prioritizing accessibility and adaptability, PlatROB reduces barriers for institutions integrating practical robotics education, offering a flexible, robust foundation for experiential learning.

However, PlatROB has limitations. The sensor suite, adequate for foundational tasks, requires enhancement for advanced perception and AI research. While compatible with the Arduino IDE, deeper integration with ROS/ROS2 for complex multi-agent systems and simulations is needed for advanced users. Additionally, scaling PlatROB to large-scale institutional deployment will also require developing comprehensive instructor training programs and technical support resources. Future improvements will include integrating advanced sensors like LiDAR and RGB-D cameras to support sophisticated perception and mapping tasks, developing ROS/ROS2 packages and digital twin simulations for virtual-physical learning experiences, expanding open-access curriculum materials, and designing specialized modules to broaden application domains. These enhancements will ensure PlatROB remains a versatile, evolving foundation for accessible robotics and AI education while maintaining its core principles of modularity, affordability, and open-source collaboration.

## Ethics statements

The authors confirm that informed consent was obtained from all student participants in the educational impact component prior to data collection (surveys administered during workshops and a summer course). Participants were informed of the study's purpose, procedures, and their right to withdraw at any time.

## Declaration of generative AI and AI-assisted technologies in the writing process

During the preparation of this work the authors used Google’s Gemini (version 2.5 Pro) and OpenAI’s ChatGPT (versions o4 and 5 Thinking). ChatGPT o4 was used for literature classification and structure organization, while Gemini and ChatGPT 5 were used for language improvement and documentation review. After using this tool/service, the authors reviewed and edited the content as needed and take full responsibility for the content of the publication.

## CRediT authorship contribution statement

**Jose Balbuena:** Writing – review & editing, Writing – original draft, Validation, Methodology, Formal analysis, Conceptualization. **Julio Sinche:** Writing – original draft, Validation, Investigation. **Diego Quiroz:** Writing – original draft, Project administration, Methodology, Investigation. **Diego Arce:** Writing – review & editing, Writing – original draft, Supervision. **Elizabeth Villota:** Writing – review & editing, Writing – original draft, Funding acquisition, Conceptualization.

## Declaration of competing interest

The authors declare that they have no known competing financial interests or personal relationships that could have appeared to influence the work reported in this paper.
